# Loss of TMEM55B modulates lipid metabolism through dysregulated lipophagy and mitochondrial function

**DOI:** 10.1038/s41419-025-08210-x

**Published:** 2026-01-09

**Authors:** Yuanyuan Qin, Sheila S. Teker, Nilsa La Cunza, Yao Tong, Elizabeth Theusch, Neil V. Yang, Leela Venkatesan, Julia Su, Xuanwen Wang, Ronald M. Krauss, Aparna Lakkaraju, Aras N. Mattis, Marisa W. Medina

**Affiliations:** 1 Department of Pediatrics, University of California San Francisco, Oakland, CA USA; 2 Department of Ophthalmology, University of California San Francisco, San Francisco, CA USA; 3 Department of Nutritional Sciences and Toxicology, University of California Berkeley, Berkeley, CA USA; 4 Liver Center, University of California San Francisco, San Francisco, CA USA; 5 Pharmaceutical Sciences and Pharmacogenomics Graduate Program, University of California San Francisco, San Francisco, CA USA; 6 Department of Pathology, University of California San Francisco, San Francisco, CA USA; 7 Institute for Human Genetics, University of California San Francisco, San Francisco, CA USA

**Keywords:** Mechanisms of disease, Endocrine system and metabolic diseases, Autophagy

## Abstract

Lipophagy is a form of selective autophagy that targets the lipid droplets for lysosomal decay and has been implicated in the onset and progression of metabolic dysfunction-associated steatotic liver disease (MASLD). Factors that augment lipophagy have been identified as targets for MASLD therapeutic development. TMEM55B is a key regulator of lysosomal positioning, which is critical for lysosome fusion with the autophagosome, but is less well studied. Here, we demonstrate that the absence of TMEM55B in murine models accelerates MASLD onset and progression to metabolic dysfunction-associated steatohepatitis (MASH). In cellular models, TMEM55B deficiency enhances incomplete lipophagy, whereby lysosome-lipid droplet interactions are increased, but lysosomal cargo is not fully degraded and/or released, leading to the development of lipid-filled lysosomes (lipolysosomes). Loss of TMEM55B also impairs mitophagy, causing an accumulation of dysfunctional mitochondria. This imbalance leads to increased lipid accumulation and oxidative stress, worsening MASLD. These findings underscore the importance of lysosomal positioning in lipid metabolism and suggest that targeting lipophagy for MASLD therapeutic development should be carefully considered to ensure promotion of the entire lipophagic flux pathway and whether it occurs in the context of mitochondrial dysfunction.

## Introduction

Lipophagy and mitophagy are forms of selective autophagy essential for maintaining cellular lipid and energy homeostasis. Lipophagy targets lipid droplets for degradation in the lysosome, where triglycerides are broken down into fatty acids [1]. Mitophagy is the selective degradation of damaged or dysfunctional mitochondria [2]. Both impaired lipophagy and mitophagy have been implicated in the development and progression of metabolic dysfunction-associated steatotic liver disease (MASLD) in human patients and pre-clinical models [3–5]. MASLD arises from excessive hepatic lipid accumulation, progressing from hepatic steatosis to metabolic-associated steatohepatitis (MASH), fibrosis, and cirrhosis [6]. Impaired lipophagy disrupts triglyceride metabolism, leading to lipid overload and hepatocellular stress [7], while defective mitophagy reduces fatty acid oxidation and promotes oxidative stress, further driving MASLD progression [5]. Rezdiffra (generic name Resmetirom), the only FDA-approved therapeutic for MASH treatment, increases lipophagy and mitophagy [8], highlighting the importance of these pathways in MASLD [9]. Notably, these processes require functional lysosomes, which play a critical role in autophagic degradation [10].

Lysosomal function depends on multiple factors, including positioning within the cell. Lysosomes are typically distributed throughout the cell; however, they must travel to the perinuclear region to fuse with autophagosomes [11]. Transmembrane protein 55B (TMEM55B), also known as phosphatidylinositol-4,5-biphosphate 4-phosphatase 1 (PIP4P1), can regulate lysosome intracellular positioning and mobility [12–14]. TMEM55B promotes dynein-dependent lysosomal retrograde trafficking by recruiting JIP4 (C-Jun-amino-terminal kinase-interacting protein 4), a dynein motor, to the lysosomal surface [12]. In fatty acid-treated hepatoma cell lines, inhibition of TMEM55B leads to perinuclear localization of enlarged, immobile lysosomes, suggesting a role in lysosomal mobility [14]. Given the established role of lipophagy and mitophagy in MASLD, we sought to investigate how TMEM55B influences these processes as well as MASLD onset and progression.

## Results

### Loss of *TMEM55B* accelerates diet-induced MASLD onset and progression in mice

**Fig. 1:**
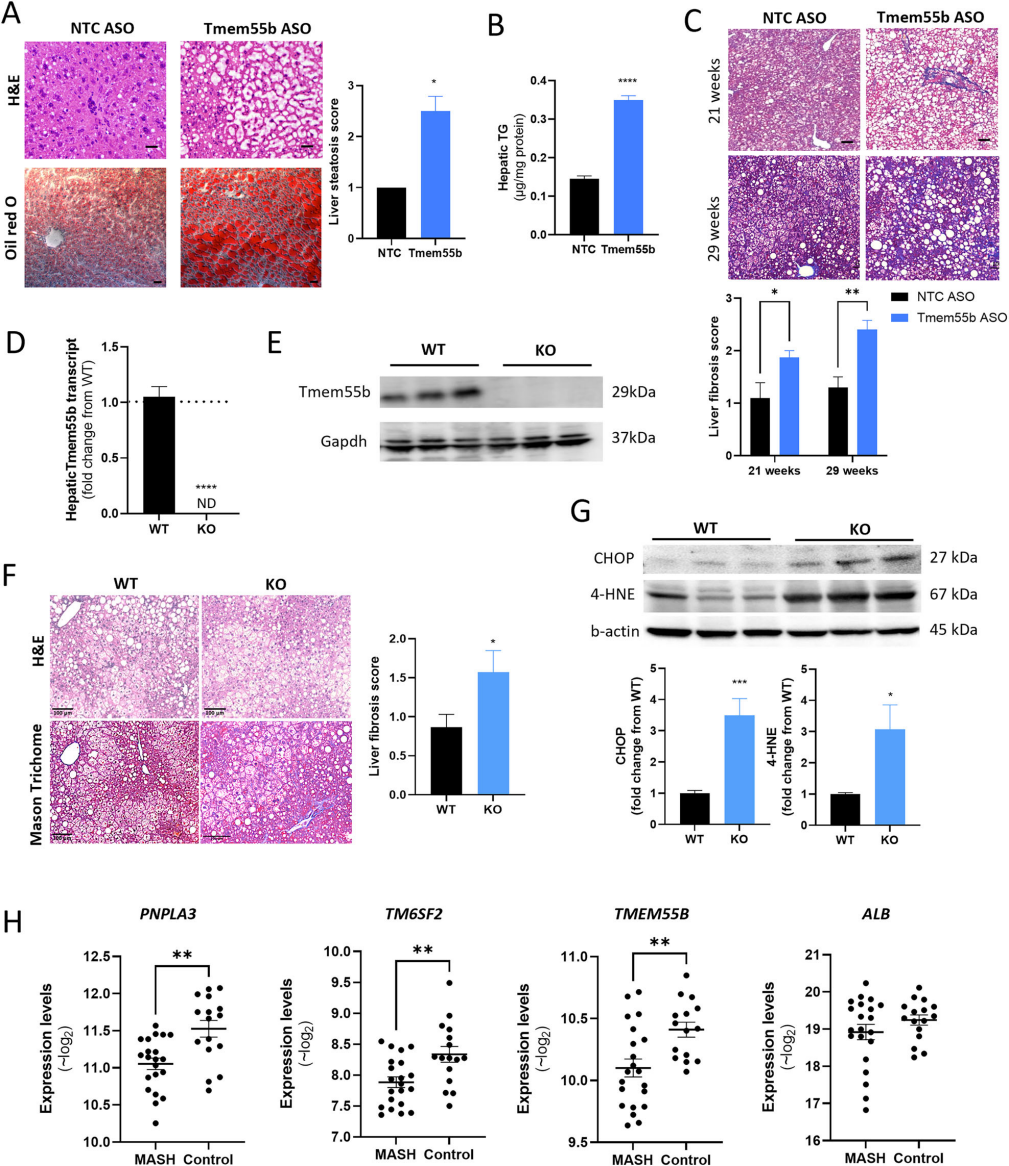
Loss of Tmem55b led to hepatic lipid accumulation and MASH development. Six-week old male C57BL/6 J mice were treated with an ASO against *Tmem55b* (Tmem55b-ASO) or a non-targeting control (NTC) at a dose of 25 mg/kg body weight/week and fed a Western diet (0.2% cholesterol, 42% fat) for 6 weeks. **A** Liver tissues were stained with Oil Red O and H&E (Scale bars = 10 µm), *N* = 2 NTC ASO, N = 4 *Tmem55b* ASO. **B** Triglyceride levels were measured in lipid extracted from the livers following Folch method, *N* = 4/ASO treatment. **C** Male C57BL/6 J mice were treated with *Tmem55b*-ASO or NTC-ASO and fed Western diet for 21 or 29 weeks. Mouse liver was stained with Masson Trichrome. Liver fibrosis was assessed by a blinded pathologist (*N* = 5 NTC ASO, *N* = 10 *Tmem55b* ASO). **D** Hepatic *Tmem55b* transcript and **E** protein levels were quantified by western blot in wildtype (WT) and *Tmem55b* KO mice(*N* = 6/genotype). Six-week-old male WT and *Tmem55b* KO mice were fed GAN diet for 12 weeks. **F** Male mouse liver was stained with H&E and Masson Trichrome. Liver fibrosis was assessed by a blinded pathologist (Scale bars=100 µm) (*N* = 7 WT, *N* = 11 KO). **G** CHOP and 4-HNE protein levels were detected by immunoblot in mouse liver tissues and quantified with Image J, *N* = 9/genotype. For animal studies, quantified results are presented as mean ± s.e.m. Representative images are shown here. *p < 0.05, **p < 0.01, ***p < 0.001, ****p < 0.0001 vs. NTC ASO or WT by one-way ANOVA or Student’s *t*-test. **H** *TM6SF2*, *PNPLA3, TMEM55B*, and *ALB* transcript levels in iPSC-Heps from NASH cases (*n* = 21) and controls (*n* = 15) quantified by RNAseq. Variance stabilized (~log2) values were calculated in DESeq and plotted. **p < 0.005. ASO antisense oligonucleotides, NTC non-target control, TMEM55B transmembrane protein 55B, Gapdh glyceraldehyde-3-phosphate dehydrogenase, CHOP C/EBP homologous protein, 4-HNE 4-hydroxynonenal, PNPLA3 patatin-like phospholipase domain-containing protein 3; TM6SF2 transmembrane 6 superfamily member 2, ALB albumin.

To assess the effect of TMEM55B inhibition in vivo, we injected Western diet-fed C57BL/6 J male mice with antisense oligonucleotides (ASOs) targeting *Tmem55b* or non-target control (NTC) (Supplementary Fig. S1A), a model that we previously showed to reduce hepatic *Tmem55b* transcript and protein levels by ~60% [14]. After 6 weeks, *Tmem55b* ASO-treated male animals had significantly greater hepatic lipid accumulation (Fig. 1A, B), with no changes in glycemic control (Supplementary Fig. S1B**)**. In C57BL/6 J male mice fed the metabolic dysfunction associated steatohepatitis (MASH)-inducing GAN diet (40% kcal fat, 20% kcal fructose, 2% cholesterol) (Supplementary Fig. S1C**),** *Tmem55b* ASO treated animals had a higher level of fibrosis after 21 and 29-weeks on diet (Fig. 1C). These findings were consistent with our RNAseq analysis where hepatic transcripts upregulated in *Tmem55b* vs. NTC ASO treated animals were enriched in cell adhesion genes (Supplementary Fig. S1D).

We also created a *Tmem55b* knockout (KO) mouse (Supplementary Fig. S1E) and confirmed the absence of hepatic *Tmem55b* transcript and protein (Fig. 1D, E). Mice were fed a GAN diet starting at 6 weeks old for 12 weeks (Supplementary Fig. S1F). Compared to *Tmem55b* flox/flox (e.g., wildtype allele, WT) littermates, both male and female *Tmem55b* KO mice had significantly increased liver fibrosis (p < 0.05) (Fig. 1F, Supplementary Fig. S2A). Male *Tmem55b* KO mice had increased liver weight (p < 0.01) and trends of greater plasma ALT and hepatocyte ballooning (Supplementary Fig. S2B). *Tmem55b* KO livers from female mice had 3-fold greater levels of ER and oxidative stress markers (Fig. 1G). Male and female *Tmem55b* KO mice had increased plasma total cholesterol and non-HDL cholesterol (Supplementary Fig. S3), consistent with previously published effects of *Tmem55b* knockdown [14].

### *TMEM55B* levels are lower in iPSC-derived hepatocyte-like cells from MASH patients compared to healthy controls

Transcript differences detected in livers from MASLD cases compared to healthy controls may be a consequence of, rather than a cause of, the disease. In contrast, differences observed in induced pluripotent stem cell-derived hepatocyte-like cells (iPSC-Heps) may reflect genetic contributors to disease. Using RNAseq data (GEO# GSE138312) from a previously described cohort of iPSC-Heps from MASH cases and healthy controls [15], we found that iPSC-Heps from MASH cases had lower levels of *PNPLA3* and *TM6SF2* (Fig. 1H), which contain loss-of-function alleles that promote MASH development [16, 17]. Similar levels of albumin (*ALB*), a hepatocyte-specific gene, indicative of equivalent differentiation efficiency, were observed between the two groups. Importantly, we found reduced *TMEM55B* transcript levels in the MASH iPSC-Heps (P < 0.01, Fig. 1H).

### *TMEM55B* knockdown impairs lysosomal activity and decreases autophagy

**Fig. 2:**
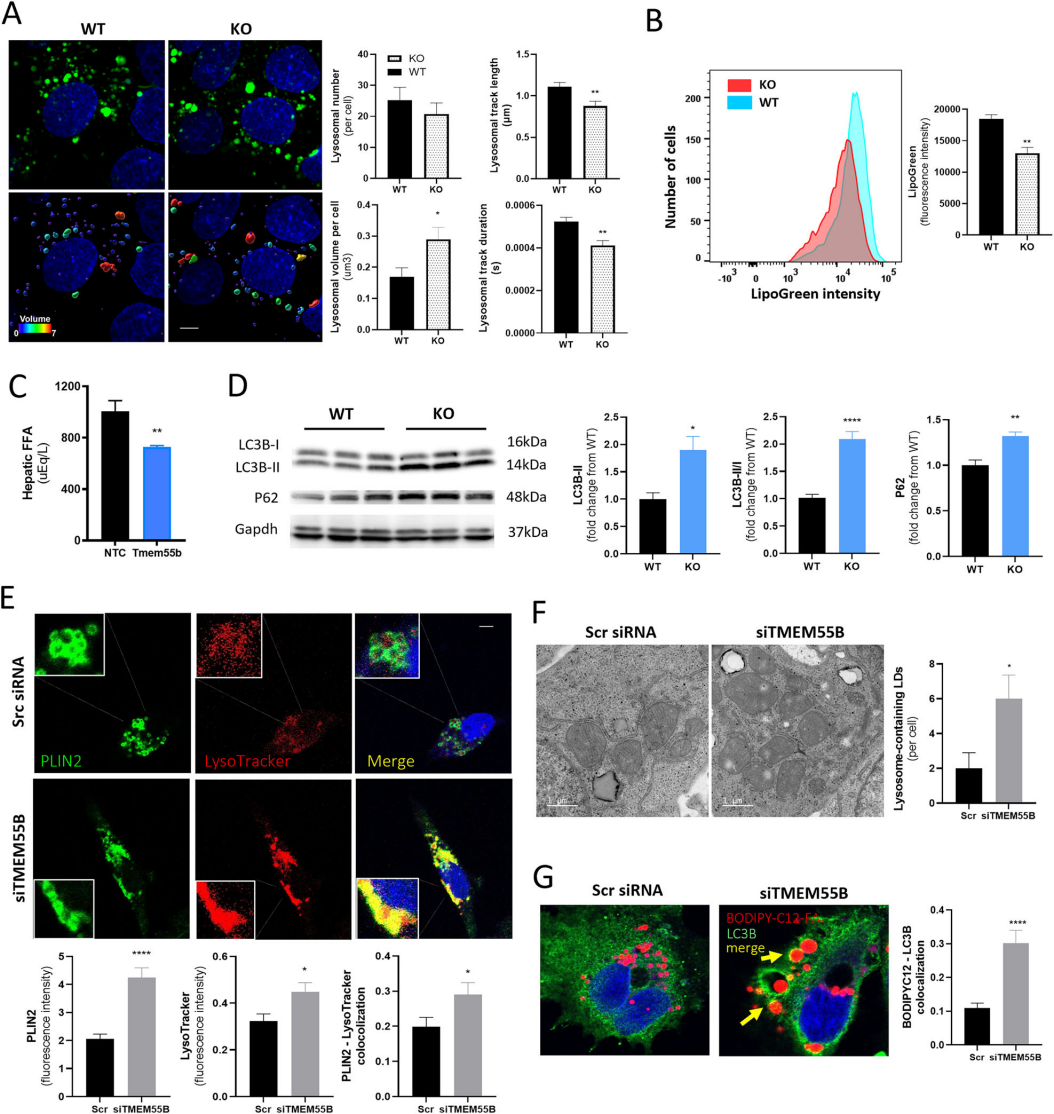
The effect of Tmem55b deficiency on lysosomes and autophagy. **A** Primary hepatocytes were isolated from one *Tmem55b* KO and one WT male littermate and treated with 1 mM oleate for 1 hour, stained with LysoTracker, and imaged on a Nikon spinning disk confocal microscope at 100X (Scale bars = 5 µm). For analysis of lysosome volume, lysosomes were subjected to surface reconstruction in Imaris, and automated segmentation by color-coding based on the volume of the connected components was used for 3D surface rendering of lysosomes (bottom panels). Representative images are shown. Six non-overlapping images were acquired/genotype and quantified. **B** Lysosomal acid lipase activity was measured on the BD LSRFortessa Cell Analyzer in mouse primary hepatocytes from *Tmem55b* KO and WT male littermates, *N* = 2/genotype. **C** Six-week-old male C57BL/6J mice were treated with an ASO against *Tmem55b* or NTC at a dose of 25 mg/kg body weight/week and fed a Western diet (0.2% cholesterol, 42% fat) for 6 weeks. Free fatty acid levels were measured in the liver tissue lysate, *N* = 8/ASO treatment. **D** Six-week-old male WT and *Tmem55b* KO mice were fed a GAN diet for 12 weeks, and p62 and LC3B-I and II protein levels were detected by immunoblot in mouse liver tissues and quantified with Image J, *N* = 6/genotype. **E** HepG2 cells were transfected with *TMEM55B* or Scr siRNAs. After 48 hr, HepG2 cells were labeled with anti-PLIN2 and LysoTracker and examined by confocal microscopy (63X, scale bars = 10 µm), *N* = 30 cells Scr siRNA, *N* = 33 cells *TMEM55B* siRNA. **F** Electron micrographs of HepG2 cells showing lysosome-containing lipid droplets (LDs) with *TMEM55B* knockdown (63X, scale bars=10 µm), *N* = 5 images Scr siRNA, *n* = 6 images *TMEM55B* siRNA. **G** HepG2 cells were transfected with *TMEM55B* or Scr siRNAs. After transfection, HepG2 cells were incubated with 1 µm of red BODIPY™ 558/568 C12-FA overnight, stained with anti-LC3B antibody and subsequently Goat anti-rabbit IgG H&L, and examined by confocal microscopy. *N* = 33 cells Scr siRNA, *N* = 37 cells *TMEM55B* siRNA. Representative images shown. Fluorescence was quantified on the BD LSRFortessa Cell Analyzer as the median fluorescence values of 10,000 gated events. Results are presented as mean ± s.e.m. *p < 0.05, **p < 0.01, ***p < 0.001, ****p < 0.0001 vs. Scr siRNA or WT by Student’s *t*-test. WT wildtype Tmem55b fl/fl mice, KO Tmem55b whole-body knockout mice, ASO antisense oligonucleotides, NTC non-target control, LC3B Microtubule-Associated Protein 1 Light Chain 3 Beta, Gapdh glyceraldehyde-3-phosphate dehydrogenase, Scr siRNA scramble small interfering RNA, LDs lipid droplets, FA fatty acid.

TMEM55B is a transmembrane protein located on late endosomes and lysosomes [18]. We previously reported that *TMEM55B* knockdown in HepG2 cells increased levels of the lysosome marker LAMP1 (lysosome-associated protein 1) and caused lysosomal clustering in the perinuclear region upon fatty acid exposure [14]. Here, we extend these observations to primary murine hepatocytes from *Tmem55b* KO mice. After incubation with 1 mM oleate for 1 hour, lysosomal volume was higher and lysosomal track length and duration were reduced in hepatocytes from *Tmem55b* KO mice, while lysosome number remained unchanged compared to WT mice (Fig. 2A**)**. Similar effects were observed upon *TMEM55B* knockdown in HepG2 cells, indicating decreased lysosome mobility (Supplementary Fig. S4A–C). *Tmem55b* KO mouse hepatocytes showed a 29% decrease (*p* < 0.01) of lysosome acid lipase activity (Fig. 2B), the enzyme responsible for hydrolyzing lysosomal triglycerides for the release of free fatty acids [19]. Reflecting this reduction in lipase activity, we observed lower hepatic free fatty acid levels after *Tmem55b* ASO-mediated knockdown (Fig. 2C). *TMEM55B* knockdown had no effect on cytosolic lipase activity in human hepatoma cell lines (Supplementary Fig. S4D). Given the importance of lysosome function in autophagy, we tested whether generalized markers of autophagy were impacted by TMEM55B. *Tmem55b* KO animals had greater hepatic LC3B-II levels (*P* < 0.0001), LC3B-II/I ratio (*P* < 0.05), and p62 levels (*P* < 0.01) compared to WT littermate controls (Fig. 2D), all indicative of reduced autophagic flux. In addition, *TMEM55B* knockdown with or without Bafilomycin A-treated HepG2 cells also led to an increased LC3B-II/I ratio (Supplementary Fig. S4E). Together, these findings suggest that loss of TMEM55B reduces lysosomal activity and impairs autophagy.

### Loss of TMEM55B promotes incomplete lipophagy

To further examine lipophagy, we tested the effect of *TMEM55B* knockdown on lysosomes and lipid droplets colocalization. In the control treated cells, the lipid droplet marker perilipin-2 (PLIN2) [20], revealed distinct circles around the periphery of lipid droplets (Fig. 2E). Upon *TMEM55B* knockdown, PLIN2 levels were increased, the peripheral circular structure was lost, and there was greater colocalization with lysosomes (1.5-fold, *p* < 0.05, Fig. 2E), indicative of the formation of lipid-filled lysosomes (aka lipolysosomes) [21]. Similar results were observed after an oleate challenge (Supplementary Fig. S4F). We confirmed this increase in lysosomal lipid accumulation (Supplementary Fig. S4G, S4H) by incubating cells with BODIPY-labeled C12-FA or C16-FA and staining with lysosome-associated membrane protein 1 (LAMP1) or LysoTracker. *TMEM55B* knockdown increased lysosome intensity levels (1.2-fold, *p* < 0.01) and colocalization of neutral lipids and lysosomes (1.5-fold, p < 0.001, Supplementary Fig. S4G, S4H). Increased lipolysosome formation with TMEM55B knockdown was confirmed in HepG2 cells using electron microscopy (Fig. 2F). We also observed changes in lipolysosome positioning. Lipolysosomes appeared primarily at the cell periphery in control cells (Supplementary Fig. S4G), versus within the perinuclear region after *TMEM55B* knockdown. Lastly, *TMEM55B* knockdown led to greater colocalization of C12-FA to LC3B (Fig. 2G). These findings indicate that loss of TMEM55B leads to greater interactions between the lipid droplet, autophagosome, and lysosome, which would typically suggest increased lipophagy. However, as this conclusion contradicts our observations of impaired lysosomal activity and autophagy, we further examined lipophagic flux.

**Fig. 3:**
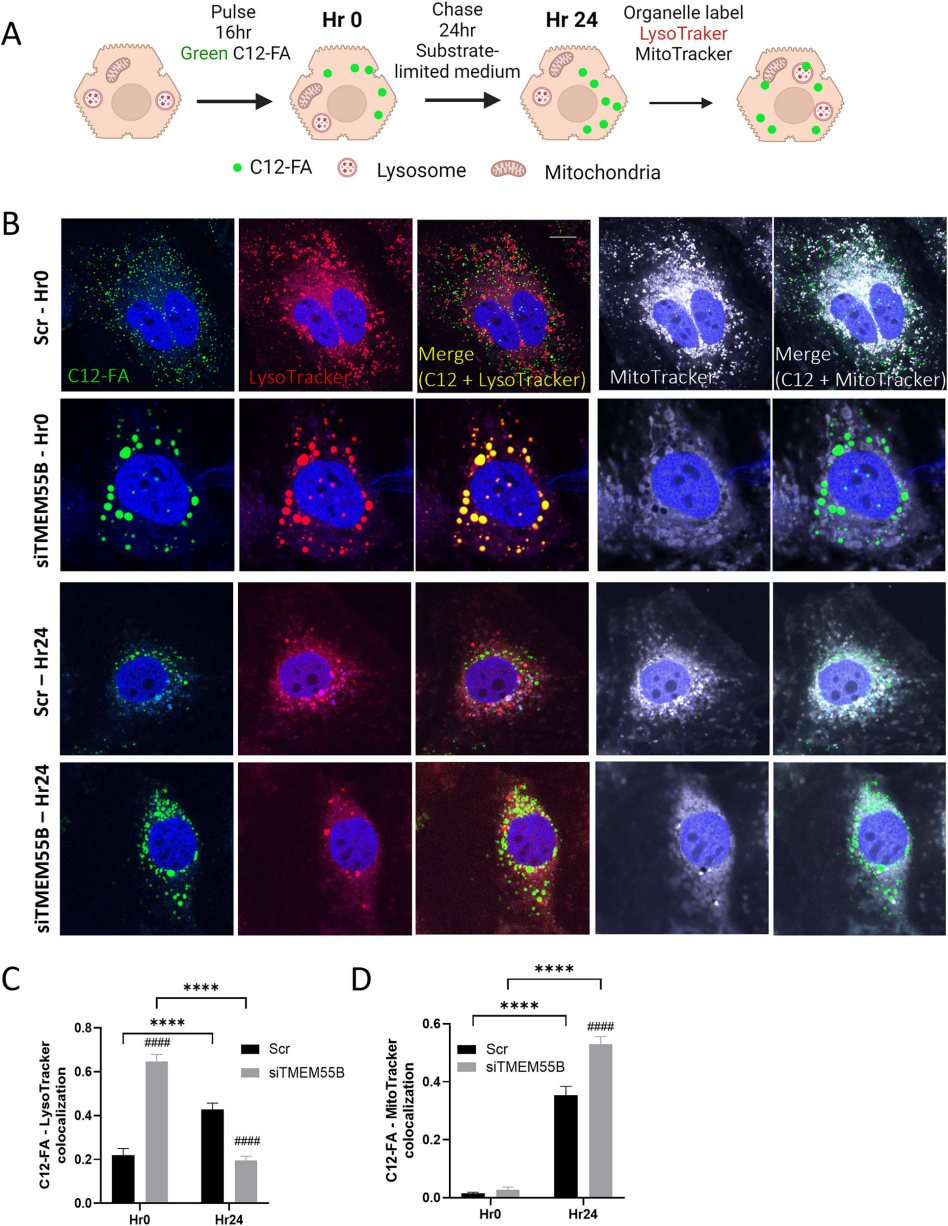
Elevated autophagy flux by *TMEM55B* knockdown increased lysosomal fatty acid release to mitochondria. **A** Schematic representation of the FA pulse-chase assays. HepG2 cells were transfected with *TMEM55B* and Scr siRNAs, pulsed with Green C12-FA (2 µM) for 16 hours, washed, and chased with substrate-limited DMEM supplemented with 0.5 mM of glucose, 1 mM of glutamine, 0.5 mM of carnitine, and 1% FBS for 24 hours. Cells were then labeled with LysoTracker Red (shown as Red), and MitoTracker Deep Red (shown as white), and imaged with Zeiss 710 confocal microscope at 63X (Scale bars=10 µm) **B** Representative images. **C** Quantification of C12-FA localization to lysosomes before (Hr0) and after (Hr24) the chase. **D** Quantification of C12-FA localization to mitochondria before (Hr0) and after (Hr24) the chase. *N* = 67 cells Scr siRNA, *N* = 69 cells *TMEM55B* siRNA at Hr0; *N* = 93 cells Scr siRNA, *N* = 79 cells *TMEM55B* siRNA at Hr24. Results are presented as mean ± s.e.m. **p < 0.01, ****p < 0.0001 vs Hr0; ###*p* < 0.001, ####*p* < 0.0001 vs Scr by two-way ANOVA. Scr siRNA scramble small interfering RNA, LDs lipid droplets, FA fatty acid.

Lipophagy is a multi-step process where lipid droplets are first engulfed by autophagosomes, fused with lysosomes, and degraded, and the resulting free fatty acids are trafficked to mitochondria for β-oxidation [22], as well as lipid droplets [23] or the outside of the cell [24]. We tested whether loss of *TMEM55B* impacts fatty acid mobilization and trafficking between mitochondria, lipid droplets, and lysosomes, using pulse-chase assays. After transfection, HepG2 cells were incubated with 2 µM BODIPY C12-FA for 16 hours to allow incorporation into neutral lipids and storage within lipid droplets, and chased with substrate-limited DMEM for 24 hours after which cells were labeled with markers for lipid droplets, lysosomes, or mitochondria (Fig. 3A, Supplementary Fig. S5A). After the 16-hour pulse (Hr 0 of chase), *TMEM55B* knockdown led to greater localization of C12-FA to lysosomes (2-fold, *p* < 0.0001, Fig. 3B, C) and lipid droplets (1.4-fold, *p* < 0.0001, Supplementary Fig. S5B), consistent with our prior observations of enlarged lipid-filled lysosomes (Fig. 2E, F, Supplementary Fig. S4G). In the control cells, after the 24-hour chase of substrate-limited media, C12-FA colocalization to lysosomes and mitochondria was increased, consistent with the known cellular response of increased fatty acid β-oxidation during starvation [25]. In addition, there was greater C12-FA colocalization to lipid droplets (Supplementary Fig. S5B), a widely reported paradoxical characteristic of hepatocytes in response to starvation [26]. However, after the 24-hour chase, *TMEM55B* knockdown led to significantly greater C12-FA colocalization to mitochondria (1.5-fold, *p* < 0.0001, Fig. 3B, D), and reduced colocalization to lysosomes (1.5-fold, *P* < 0.0001, Fig. 3B, C). As the loss of Tmem55b reduces lysosomal acid lipase activity (Fig. 2B), the increased C12-FA containing particles delivered to mitochondria may include partially digested lipids and free fatty acids.

### *TMEM55B* knockdown prevents mitophagy, leading to mitochondrial dysfunction and oxidative stress

**Fig. 4:**
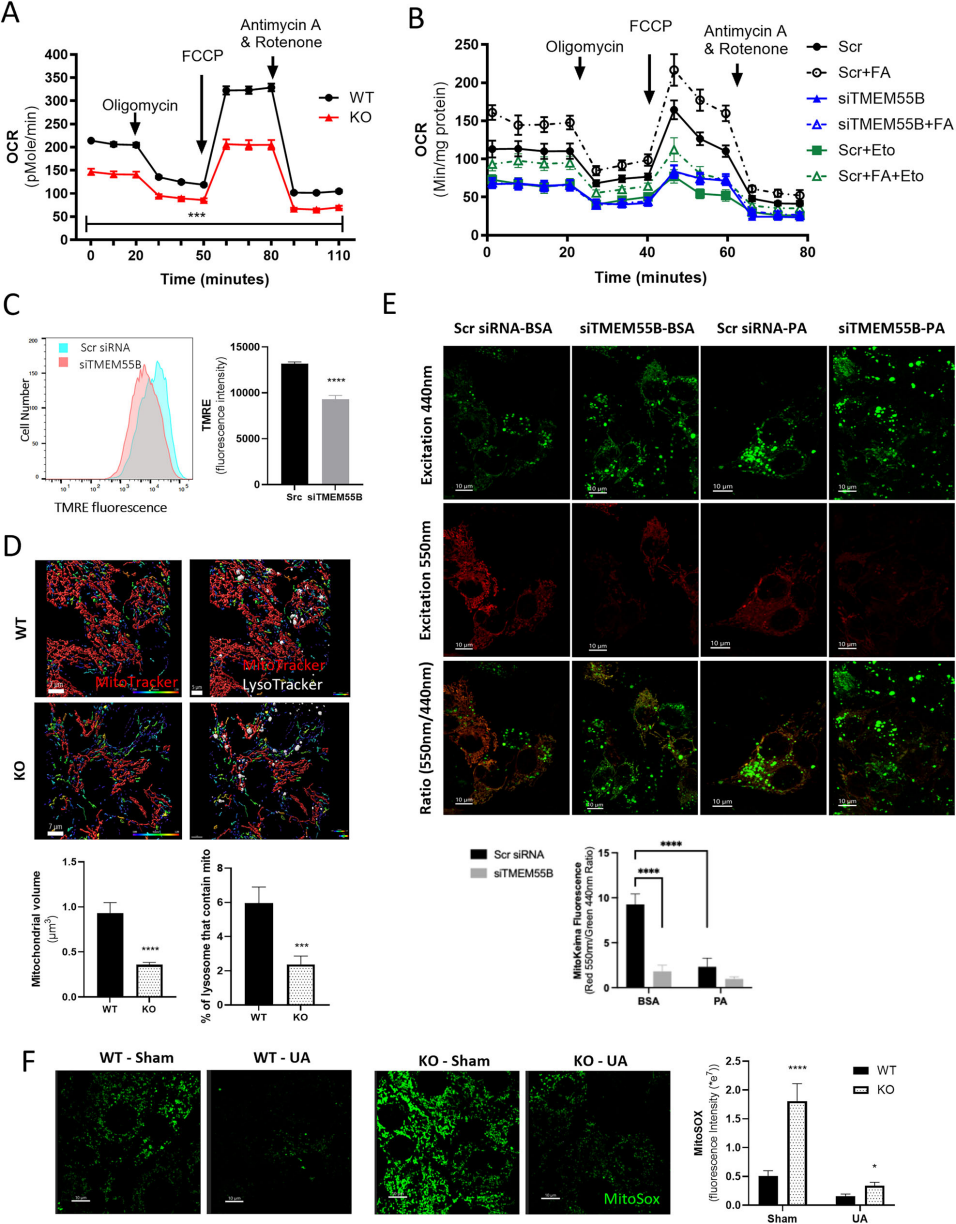
Loss of TMEM55B causes mitochondrial dysfunction, increased oxidative stress, and decreased mitophagy. **A** Oxygen consumption rate (OCR) of primary hepatocytes from one female *Tmem55b* KO (*N* = 32 technical replicates/genotype) and one female WT mouse (*N* = 30 technical replicates). **B** HepG2 cells were transfected with siRNAs targeting *TMEM55B* or scrambled siRNA and OCR was measured by Seahorse XF Analyzer with and without 0.125 mM palmitic acid (FA) and/or 40 µM etomoxir (Eto), *N* = 6 technical replicates/siRNA. **C** siRNA-transfected HepG2 cells were incubated with 50 nM TMRE for 25 minutes to stain mitochondrial membrane potential, and fluorescence was quantified in 10,000 gated events on the BD LSRFortessa Cell Analyzer. N = 5 technical replicates Scr siRNA, N = 12 technical replicates *TMEM55B* siRNA. **D** Primary hepatocytes were isolated from one male WT and one male *Tmem55b* KO mouse, incubated with MitoTracker Deep Red (shown as red) and LysoTracker Red DND-99 (shown as white), and visualized using live cell imaging on a Nikon spinning disk confocal microscope at 100X (Scale bars=7 µm). In these images, red indicates healthy mitochondria, while unhealthy mitochondria are observed as blue or purple. Images were subjected to Gaussian filtering and background subtraction in Imaris v9.6. Mitochondria were subjected to surface reconstruction in Imaris, and automated segmentation by color-coding based on the volume of the connected components was used for 3D surface rendering of mitochondria. Non-overlapping images from WT (*N* = 13) and KO (*N* = 19) were quantified. **E** HepG2 cells were transfected with MitoKeima plasmid and siRNAs targeting *TMEM55B* or scramble siRNA (Scr) using the AMAXA Nucleofector. After 48 hours, cells were treated with BSA or 1 mM PA for 1 hour and imaged and quantified on a Nikon spinning disk confocal microscope at 60X. N = 5 images/siRNA. **F** Primary hepatocytes from one female *Tmem55b* KO and one WT mouse were treated with DMSO (sham) or 20uM Urolithin A (UA) for 16 hours, followed by 1 mM palmitate (PA) for 1 hour. Cells were then incubated with 5 µM MitoSox and imaged at 60X on Nikon spinning disk confocal microscope. *N* = 5 non-overlapping images/genotype (Scale bars=10 µm). Representative images are shown. Results are presented as mean ± s.e.m. *p < 0.05, **p < 0.01, ***p < 0.001, ****p < 0.0001 vs. Scr or WT by Student’s *t*-test or 2-way ANOVA. WT wildtype Tmem55b fl/fl mice, KO Tmem55b whole-body knockout mice, Scr siRNA scramble small interfering RNA, FA fatty acid, ETO etomoxir,TMRE Tetramethylrhodamine ethyl ester, BSA bovine serum albumin, PA palmitate, DMSO dimethyl sulfoxide, UA urolithin a.

Excessive fatty acid trafficking to mitochondria, particularly in the form of lipid intermediates, can lead to mitochondrial damage [27]; thus, we tested TMEM55B effects on mitochondrial function. Primary hepatocytes from female *Tmem55b* KO mice showed dramatically reduced mitochondrial oxygen consumption rate (OCR) (Fig. 4A) compared to WT littermate controls. Basal respiration, ATP production, maximal respiration, non-mitochondrial respiration, proton leak, and spare capacity were all significantly decreased (Supplementary Fig. S6A). Similar reductions in mitochondrial activity were observed in HepG2 cells with *TMEM55B* knockdown (Fig. 4B, Supplementary Fig. S6B). While the addition of exogenous fatty acids normally increases both basal and maximal OCR, as seen in the control-treated cells, there was no change in OCR upon palmitate addition after *TMEM55B* knockdown (Fig. 4B), even in permeabilized cells (Supplementary Fig. S6C). We observed similar effects in mitochondria isolated from the livers of male mice treated with *Tmem55b* ASO (Supplementary Fig. S6D), which together indicate that loss of TMEM55B prevents utilization of exogenous fatty acid for mitochondrial β-oxidation.

To determine whether the reduction in mitochondrial fatty acid β-oxidation could be due to impaired mitochondrial function or/and reduced levels of mitochondria, we evaluated the effects of *TMEM55B* knockdown on mitochondrial membrane potential, essential for the production of ATP [28]. *TMEM55B* knockdown reduced TMRE (tetramethylrhodamine ethyl ester, a dye that accumulates only in the membrane of active mitochondria) levels by 35% in HepG2 cells (*p* < 0.05, Fig. 4C). We also observed reduced mitochondrial volume by 61% with 3D-live cell imaging using MitoTracker Deep Red in primary hepatocytes from male *Tmem55b* KO mice and WT controls (*p* < 0.0001, Fig. 4D), and decreased mitochondria intensity by 42% in HepG2 cells stained with MitoGreen (*p* < 0.001, Supplementary Fig. S6E). Using live cell imaging with 3D reconstruction, we observed highly fragmented mitochondrial networks, a sign of mitochondria dysfunction [29], in primary hepatocytes from *Tmem55b* KO mice (Fig. 4D). *Tmem55b* KO led to a significantly lower percentage of lysosomes containing mitochondria (*p* < 0.001, Fig. 4D), while knockdown caused a 28% decrease in LC3B and MitoTracker colocalization (*p* < 0.05, Supplementary Fig. S6F), indicative of impaired mitophagy. We confirmed that *TMEM55B* knockdown led to a drastic (>80%) reduction in mitophagy using the MT-mKeima-Red assay to directly visualize mitophagy (*p* < 0.0001, Fig. 4E, Supplementary Fig. 7).

Mitochondrial damage often leads to oxidative stress, and not surprisingly, *TMEM55B* knockdown led to significantly higher levels of oxidative stress using a generalized measure of oxidative stress and specific measures of mitochondrial oxidative stress (Supplementary Fig. S6G, S6H). Importantly, palmitate (1 mM) induced significantly higher mitochondria and total cellular oxidative stress *Tmem55b* KO primary hepatocytes compared to WT (Fig. 4F, Supplementary Fig. S6H, S6I), an effect that was mitigated upon incubation with 20 µM urolithin A, which stimulates mitophagy and maintains mitochondrial function [30, 31]. Together, these findings suggest that loss of *TMEM55B* results in mitochondrial dysfunction through impaired mitophagy.

### *TMEM55B* knockdown increases FA uptake and causes cellular steatosis through lysosome positioning

**Fig. 5:**
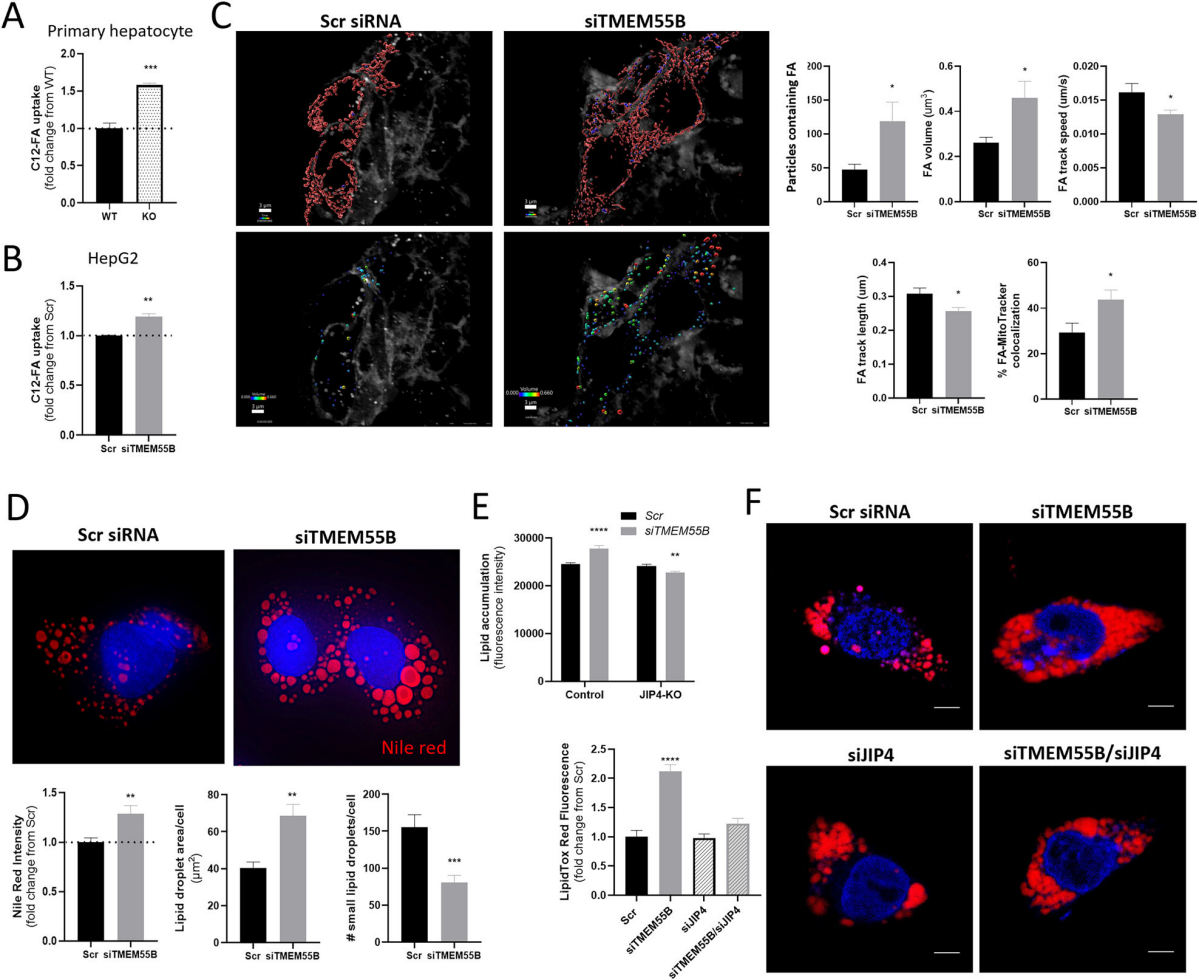
*Tmem55b* knockdown regulates cellular lipids through the lysosome position. **A** Primary hepatocytes isolated from one male *Tmem55b* KO and one male WT mouse were incubated with 2 µm BODIPY C12-FA for 30 min, and fluorescence was quantified by the BD LSRFortessa Cell Analyzer in 10,000 gated events, N = 4 technical replicates/genotype. **B** HepG2 cells were transfected with siRNA targeting *TMEM55B* or a scrambled control, and after 48 hr treated with 2 µm BODIPY C12-FA for 30 mins. Fluorescence was quantified by flow cytometry as described above, *N* = 3 technical replicates/treatment. **C** Transfected HepG2 cells were incubated with 2 µm BODIPY C12-FA for 30 mins before live cell imaging with Nikon spinning disk confocal microscope at 100X (Scale bars=3 µm), *N* = 10 non-overlapping images Scr siRNA, *N* = 12 images *TMEM55B* siRNA. Mitochondria and FAs were subjected to surface reconstruction in Imaris. Volume of FA containing particles is indicated by color. **D** Huh7 cells were transfected with *TMEM55B* and Scr siRNAs, incubated with 1 mM oleate for 24 hr, stained with Nile red, and visualized using confocal microscopy (Scale bars=10 µm), *N* = 10 cells Scr siRNA, *N* = 15 cells *siTMEM55B* siRNA. **E** Control and *JIP4*-knockout (KO) HepG2 cells were transfected with siRNAs targeting *TMEM55B* or scrambled control, incubated with 200 ng/ml BODIPY493/503, and the fluorescence was quantified by the BD LSRFortessa Cell Analyzer in 10,000 gated events, *N* = 4 technical replicates/siRNA. **F** HepG2 cells were transfected with siRNAs targeting *TMEM55B* (*siTMEM55B*) or JIP4 (siJIP4) individually or together, or a scrambled control siRNA. After 48 hours, cells were incubated with 1 mM oleate for 24 hours, stained with LipidTOX Red, and imaged with Zeiss 710 confocal microscope at 63X (Scale bars=10 µm), *N* = 32 cells Scr siRNA, N = 59 cells *TMEM55B* siRNA, *N* = 44 cells JIP4 siRNA, *N* = 41 cells *TMEM55B/JIP4* siRNAs. Representative images are shown. Results are presented as mean ± s.e.m. *p < 0.05, **p < 0.01, ***p < 0.001, ****p < 0.0001 vs. Scr by Student’s *t*-test. WT wildtype Tmem55b fl/fl mice, KO Tmem55b whole-body knockout mice, Scr siRNA scramble small interfering RNA, JIP4 C-Jun-amino-terminal kinase-interacting protein 4, also known as SPAG9.

Given the inability of cells to utilize exogenous FA for mitochondrial β-oxidation in the absence of TMEM55B, we tested whether there were defects in FA uptake. There was a greater FA uptake after *Tmem55b* KO in primary murine hepatocytes and *TMEM55B* knockdown in HepG2 cells after incubation with BODIPY-C12 for 30 minutes (Fig. 5A, B). *TMEM55B* knockdown led to greater number and volume/cell of FA containing particles, as well as reduced motility and track length of these particles (Fig. 5C). Notably, exogenous fatty acids appeared to have greater trafficking to the mitochondria as there was a 1.5-fold increase in FA-mitochondria colocalization (*p* < 0.05, Fig. 5C). There were no differences in protein levels of CD36, a FA transporter, in the livers between ASO-*Tmem55b* and ASO-NTC treated male or female mice (Supplementary Fig. S8A).

The lack of *Tmem55b* led to hepatic steatosis in vivo (Fig. 1A–C), an effect we confirmed after *TMEM55B* knock-down in Huh7 cells (Supplementary Fig. S8B), incubated with 1 mM oleic acid and stained cells with Nile Red, a neutral lipid dye. After 24 hours there was 29% greater Nile red staining, 70% larger lipid droplet area per cell (*p* < 0.01, Fig. 5D), and fewer smaller lipid droplets with *TMEM55B* knockdown (*p* < 0.001, Fig. 5D), which is consistent with reports that only smaller lipid droplets can be engulfed by lysosomes through lipophagy [32]. Similar effects were observed in HepG2 cells incubated with BODIPY C12-FA (Supplementary Fig. S8C, S8D) and in *Tmem55b* KO primary murine hepatocytes, an effect that was enhanced after incubation with 1 mM palmitate and partially rescued with urolithin A (Supplementary Fig. S8E).

Given the role of TMEM55B in lysosome motility [12, 14, 33], which we confirmed here in HepG2 cells (Supplementary Fig. S4C), we tested whether the observed cellular steatosis was dependent on the ability of TMEM55B to modulate lysosome movement. TMEM55B enables lysosome transport by recruiting JIP4, a dynein scaffold, to the lysosome [12]. We created a HepG2 *JIP4* knockout (KO) cell line using CRISPR-Cas9, which led to an 86% reduction in *JIP4* transcript levels compared to HepG2 cells treated with a non-targeting control (NTC) gRNA (Supplementary Fig. S8F). While *TMEM55B* knockdown led to increased lipid accumulation in the NTC gRNA-treated cells incubated with BODIPY C12-FA for 24 hours, this increase was not observed in the *JIP4*-KO cell line (Fig. 5E). A similar lack of lipid accumulation was also seen after *TMEM55B/JIP4* siRNA-mediated double knockdown in HepG2 cells using confocal microscope and flow cytometry (Fig. 5F, Supplementary Fig. S8G)

## Discussion

In this study, we defined a new role for TMEM55B in mediating lipid metabolism and promoting the development of MASLD through impaired selective autophagy. First, we discovered that loss of hepatic TMEM55B led to excess hepatic lipid accumulation and greater development of fibrosis in murine *Tmem55b* knockdown and KO mouse models. These findings were consistent with our observations that iPSC-derived hepatocyte-like cells from MASH patients had reduced *TMEM55B* transcript levels compared to healthy controls. Second, to investigate the mechanism by which TMEM55B deficiency accelerates MASLD onset and progression to MASH, we performed a series of ex vivo and in vitro studies which support the model that by preventing binding with the adaptor protein JIP4, responsible for facilitating lysosome movement, loss of TMEM55B increased incomplete lipophagic flux and promoted delivery of FA-containing particles to mitochondria, resulting in mitochondrial damage. This effect is exacerbated by impaired mitophagy, which disrupts overall mitochondrial health, leading to reduced FA β-oxidation and increased oxidative and ER stress. Together, these findings demonstrate how impaired lysosome trafficking can trigger a cascade of metabolic defects via disruptions in lipophagy and mitophagy, resulting in the development of MASLD/MASH.

### Absence of TMEM55B disrupts lipophagic flux and mitophagy

Lysosomes normally fuse with endosomes or autophagosomes in the perinuclear region [34], after which lysosomal hydrolytic enzymes degrade the cargo [35]. We previously reported that in human hepatoma cell lines incubated with oleic acid, *TMEM55B* knockdown led to enlarged perinuclear lysosomes [14]. Here, we show that these lysosomes are filled with lipids. Lipolysosome formation could be attributed to increased delivery of lipids to the lysosome, impaired degradative activity within the lysosome, and/or impaired release of lysosomal contents. Loss of TMEM55B led to greater colocalization of lipids with LC3B, consistent with increased macrolipophagy, an autophagosome-mediated process [36], as well as greater colocalization and direct contacts between lipid droplets and lysosomes, indicative of greater microlipophagy, an autophagosome-independent process of direct lipid extrusion from lipid droplets to lysosomes [37]. We also observed reduced lysosomal acid lipase activity, the enzyme that degrades triglycerides into free fatty acids [19], suggesting that impaired lysosomal function also contributes to lipolysosome formation. Our pulse-chase assays demonstrated greater movement of C12-FA-containing particles from the lysosome to the mitochondria. As TMEM55B is critical for the repair of lysosomes [38], this trafficking may be attributed to dysfunctional lysosomes, allowing the release of partially degraded lipid intermediates instead of free fatty acids. These findings suggest that despite increased lipid delivery to the lysosome, impaired lysosomal acid lipase activity upon loss of TMEM55B disrupts lipophagic flux.

Loss of TMEM55B reduced the number and size of mitochondria, generated fragmented mitochondrial networks, decreased mitochondrial membrane potential, and dramatically diminished mitochondrial β-oxidation activity. This is likely due to both increased mitochondrial damage as well as reduced repair. Our findings suggest that TMEM55B knockdown/knockout leads to the delivery of partially degraded lipids (and potentially toxic lipid intermediates) to mitochondria, which can cause mitochondrial damage [39, 40]. These damaged mitochondria cannot be efficiently removed due to the impairment of mitophagy. While the loss of TMEM55B appears to disrupt lipophagy at the point of degradation within the lysosome, mitophagy appears to be inhibited at a much earlier stage based on reduced interactions between mitochondria with autophagosomes or lysosomes. Urolithin A, which promotes mitophagy through the recruitment of autophagosomes to mitochondria [41], was able to rescue the increase in palmitate-induced mitochondrial oxidative stress and lipid accumulation observed in the absence of TMEM55B.

It is notable that TMEM55B knockdown decreases LC3B-mitochondrial colocalization, while simultaneously increasing LC3B colocalization with lipid droplets. Although the mechanism underlying this shift in autophagosome targeting is unclear, TMEM55B depletion has been reported to mimic a fasted state [13]. Under these conditions, cells may prioritize mobilization of free fatty acids from lipid droplets to meet immediate energy demands, leading to preferential autophagosome engagement with lipid droplets over damaged mitochondria. However, since loss of TMEM55B also impairs lipophagic flux, this could hinder recycling of autophagy machinery (including the lysosome) and reduce the availability of autophagosomes to target mitochondria. This possibility is consistent with the known role of TMEM55B as a molecular sensor that links autophagic flux and lysosomal repair during oxidative stress [38].

### Contextualizing TMEM55B effects in human disease

Our finding that loss of TMEM55B disrupts lipophagic flux and mitophagy and promotes MASLD is consistent with their established roles in MASLD. However, our observations highlight the importance of monitoring the entire lipophagic process, and not discrete measures. As the absence of TMEM55B increases lipid droplet-lysosome colocalization and causes greater delivery of FA-containing particles first to the lysosome and subsequently to the mitochondria, these results could be interpreted as increased lipophagy, which should be protective against MASLD. However, due to the impaired ability of lysosomes to degrade lipid cargo, TMEM55B knockdown/knockout ultimately causes lipid accumulation and promotes MASLD. Notably, excess lipolysosomes have been documented in MASLD patients [21, 42], where lipolysosome formation is positively correlated with the MASLD activity score and fibrosis stage [43]. The impact of TMEM55B loss on MASLD is further promoted by the disruptions in mitophagy, leading to severely disrupted mitochondrial networks, ultimately causing oxidative and ER stress. Stimulating mitophagy was able to reverse FA-induced oxidative stress seen with loss of TMEM55B, underscoring the likelihood that the more severe MASH observed in our murine models of *Tmem55b* knockdown and KO is attributed to impaired mitochondrial health [44–46].

TMEM55B impacts cellular processes that are critical for many aspects of human health beyond MASLD. For example, lipolysosomes formation, accumulation of partially degraded lipids, and impaired mitochondrial turnover are features associated with lysosomal lipid storage, neurodegenerative, and atherosclerotic diseases, and TMEM55B-mediated disruptions in autophagy and mitochondrial quality control could contribute to the pathophysiology of these conditions. Given our prior findings that loss of TMEM55B increases plasma non-HDL cholesterol [14, 47], these effects may act synergistically to promote atherosclerosis. Thus, TMEM55B may represent a broader regulatory node linking lipid metabolism, autophagic flux, and organelle homeostasis in diverse pathologic settings.

## Conclusion

Here, we demonstrate that loss of *TMEM55B* led to MASLD onset and progression to MASH through impaired lipophagic flux and mitophagy. Our findings suggest that when mitochondrial function is impaired, increased delivery of lipids to the lysosome promotes rather than ameliorates MASLD. These findings illustrate the importance of maintaining mitochondrial function and querying the entire lipophagic pathway when evaluating MASLD therapeutic strategy.

## Materials and methods

Detailed Materials and Methods are available in the Supplemental Materials.

### Quantitative real-time PCR

All assays were performed in triplicate using 100 ng cDNA on an ABI PRISM 7900 Sequence Detection System using TaqMan and SYBR Green qPCR assays.

### Western blot analyses

Protein expression levels were measured by immunoblot as described [14].

### Cell culture and transfections

Cell culture and transfection were performed as previously described [14].

### Flow cytometry

For all assays, fluorescence intensity was quantified by the BD LSRFortessa™ Cell Analyzer as the median fluorescence values of 10,000 gated events.

### Fluorescent FA Pulse-Chase

Cells were incubated with 1 mM BODIPY 558/568 C12 (Red C12, D3835, Invitrogen) or 2 mM BODIPY FL C12 (Green, D3822, Invitrogen) in duplicate wells. After 16 hr, one well was collected as timepoint “HR 0” and the other was chased with DMEM and collected after 24 hr as timepoint “HR 24”. Mitochondria were labeled with 100 nM MitoTracker Deep Red FM for 30 min, LDs were labeled with 1 µM BODIPY 493/503 for 30 min, and lysosomes were labeled with 75 nM LysoTracker Red DND-99 for 1 hr. Cells were imaged with the Zeiss LSM 710 by confocal microscopy.

### Live cell imaging

Cells were transfected with mKeima-Red-Mito-7 (Plasmid #56018, Addgene) to monitor mitophagy in live cells. Cells were incubated with 0.2 μM LysoTracker (DND-99, Life Technologies) for 15 min, 1 μM BODIPY C12 (Green, Life Technologies) for 30 minutes, 0.2 μM MitoTracker Deep Red (Life Technologies) for 15 minutes, and 5 µM MitoSOX Green for 10 minutes, and imaged on a spinning disk confocal microscope.

### Measures of mitochondrial function

Mitochondrial oxygen consumption rate (OCR) was measured on a Seahorse Bioscience XFe96 extracellular flux Analyzer as described [48].

### Animal studies

Animal studies were performed with approval from the Institutional Animal Care and Use Committee at the University of California, San Francisco, cared for in accordance to the recommendations of the Panel on Euthanasia of the American Veterinary Medical Association, and housed in AAALAC-accredited facilities. Animal studies comply with the ARRIVE guidelines.

### *Tmem55b* knockout and knockdown mouse models

*Tmem55b* floxed mice were created by inserting *loxP* sites flanking *Tmem55b* exons 1 to 6 in a C57BL6/N background. The *Tmem55b*^*fl/+*^ mice were backcrossed to the C57BL/6 J strain and crossed with Sox-2-Cre mice (Strain #:008454, JAX) to generate *Tmem55b*^*fl/-*^ mice. These mice were intercrossed to generate whole-body *Tmem55b* knockout (KO) and littermate *Tmem55b* flox/flox control (referred to in figures as “wildtype allele” or “WT”). We have previously described an ASO-mediated *Tmem55b* knockdown mouse model [14]. Six-week-old animals were randomly selected for i.p. injection with 25 mg/kg body weight/week of ASO targeting *Tmem55b* or a non-targeting control (Ionis Pharmaceuticals) and fed a GAN Diet (n = 10/treatment). Mouse liver was embedded in Tissue-Tek or paraffin, sectioned, and stained in Oil Red O solution, H&E, Sirius Red, and Masson Trichrome. Slides were assessed by a pathologist blinded to the sample identity.

### RNA-sequencing

RNAseq libraries were prepared from hepatic RNA from mice and sequenced. Sequence fragments were aligned to the mouse GRCm39 genome and GENCODE transcriptome, adjusted for library size, and analyzed using DESeq2 [49]. RNAseq data from iPSC-derived hepatocyte-like cells were downloaded from GEO (GSE138312) from a previously described MASLD cohort [15]. Gene expression count data were adjusted for library size and variance stabilized.

### Statistics

For ex vivo and in vitro experiments, representative results are reported. Data are shown as mean ± standard error of the mean (SEM). Grubb’s test for outliers was used to identify statistical outliers. Continuous variables for two groups were compared using Student’s t-tests. Continuous variables for more than two groups were compared using one-way analysis of variance (ANOVA) with Tukey’s post hoc test. Analyses were performed using GraphPad Prism 7 software (GraphPad Software, Inc. La Jolla, CA, USA). P values < 0.05 were considered statistically significant. No statistical analysis was employed to determine sample sizes. Sample sizes were established according to previous studies or our experimental experience.

## Supplementary information

Supplementary Figures

Supplementary methods

## Data Availability

RNA-Seq data from the mouse study is available at NCBI GEO# GSE273884. RNAseq data from a cohort of iPSC-Heps from biopsy-defined MASH cases and healthy controls is available at NCBI GEO# GSE138312.
