# Supplementary material for: Loss of TMEM55B modulates lipid metabolism through dysregulated lipophagy and mitochondrial function
Source: Cell Death Dis. 2026 Jan 9;17(1):26. doi: 10.1038/s41419-025-08210-x (PMC12789068; doi:10.1038/s41419-025-08210-x)
Supplement: Supplementary file 2 — Supplementary methods [file 41419_2025_8210_MOESM2_ESM.docx]

**DETAILED MATERIALS AND METHODS**

**Quantitative real-time PCR**

RNA was extracted from mouse primary hepatocytes, livers, skeletal muscles, or adipose tissues, and reverse transcribed into cDNA as previously described(1). All assays were performed in triplicate using 100ng cDNA on an ABI PRISM 7900 Sequence Detection System using TaqMan and SYBR Green qPCR assays. *TMEM55B* was quantified using a Taqman primer (Hs00292741 for human and Mm01319582_m1 for mouse) from Thermo Fisher Scientific, and *JIP4* SYBR Green primers are as follows (Forward: GGCGGCTCGAGAAAATCCGTTCTA; Reverse: AATGCGGCCGCAACTCAATCAAC). Real-time PCR results were normalized to *CLPTM* (Hs00171300, *Clptm* (Mm00524760_m1), Thermo Fisher Scientific, Waltham, MA) as an internal control.

**Western blot analyses**

Tissues were homogenized in RIPA lysis buffer with Protease Inhibitor Cocktail (Thermo Fisher Scientific), centrifuged for 15 min at x16,000g at 4°C, and the supernatants were collected. Samples were eluted with Laemmli buffer and denatured at 95°C for 5 min before loading to 8% or 4-20% Tris-Glycine gels (Thermo Fisher Scientific). After transferring, PVDF membrane was incubated with anti-Tmem55b, anti-CHOP, anti-4-HNE, anti-LC3B, anti-p62, anti-bactin, anti-CD36, or anti-Gapdh antibody at 4°C overnight, washed 3 times, and incubated with secondary antibody for 1 hr at room temperature. Enhanced chemiluminescence substrate (Thermo Fisher Scientific) was used for protein detection. Quantitative analysis of protein bands was performed using Image J (NIH, Bethesda, MD). See the Major Resources Tables for the detailed antibody information.

**Cell culture and transfections**

HepG2 or Huh7 cells were grown at 37°C and 5% CO2 in Eagle's Minimum Essential Medium (EMEM) (ATCC, Manassas, VA) supplemented with 10% FBS (HyClone, Logan, Utah), 500 U/mL penicillin/streptomycin, and 2 nmol /L GlutaMAX (Invitrogen). *TMEM55B* and/or *JIP4* knockdown was achieved by transfection of 80,000 HepG2 cells/well in 12-well plates using either siRNAs (Life Technologies) targeting *TMEM55B* (S40499), *JIP4* (S17233) or scramble siRNA (Scr) (4390843) as non-targeting control using Lipofectamine RNAimax (Invitrogen) with 20 nM siRNA for 48 hours as previously described(2). For double knockdown, siRNAs targeting *TMEM55B* and *JIP4* were added at the same time. Cellular phenotypes were quantified 48 hours post-transfection.

**Creation of *JIP4* Knockout (KO) cells**

To create *JIP4* KO cell lines, single gRNA targeting *JIP4* (VB211203-1192efm, Supplemental Fig.S9A) and non-targeting gRNA as negative control (VB220208-1241bcc, Supplemental Fig.S9B) were designed and purchase from VectorBuilder (Chicago, IL). Cas9 2NLS nuclease was purchased from Synthego (Redwood City, California). Cells were transfected with 5 μg negative control or JIP4-Cas9/sgRNA complex using Cell Line Nucleofector™ Kit T (VCA-1002, Lonza) with Amaxa Biosystems Nucleofector II. After transfection, cells were grown in media with 500 µg/ml G418 for 10 days, and 200 µg/ml G418 for another 4 days to select for plasmid expressing cells.

**Flow cytometry**

Lysosomal acid lipase activity was quantified using the LysoLive™ Lysosomal Acid Lipase Assay Kit (ab253380, Abcam). Cells were incubated with 1X LipaGreen^TM^, a substrate that becomes fluorescent upon cleavage by lysosomal acid lipase, according to the manufacturer's protocol". To measure mitochondrial membrane potential, HepG2 cells were harvested with 0.05% Trypsin-EDTA and resuspended in PBS with 50 nM tetramethylrhodamine ethyl ester (TMRE, T669, Invitrogen) for 25 minutes at 37°C and 5% CO2. To measure exogenous fatty acid uptake, cells were incubated with 200 ng/ml BODIPY493/503 (D3922, Invitrogen), 1 µm BODIPY-labelled C12- (D3822, Invitrogen), 2 µm BODIPY™ 558/568 C12 (D3835, Invitrogen) or 1 µm C16-FA (D3821, Invitrogen) for 30 mins, washed 3 times with PBS, harvested with 0.05% Trypsin-EDTA. To quantify total cellular oxidative stress, primary hepatocytes were incubated with or without 20uM Urolithin A (UA) for 16 hours, followed by BSA or 1mM palmitate (PA) treatment for 1 hour, stained with 500nM CellRox Green for 1 hr at 37°C and 5% CO2 before FACS. To quantify cellular lipid accumulation in primary hepatocytes, cells were treated with UA for 16 hours, then BSA or 1mM PA for 1 hour, stained with 200 ng/ml for BODIPY 493/503 for 30min at 37°C and 5% CO2 before FACS.

For all assays, fluorescence intensity was quantified by the BD LSRFortessa Cell Analyzer as the median fluorescence values of 10,000 gated events.

**Cellular Lipase assay**

Cellular lipase activity was measured using the Lipase Assay Kit (Colorimetric) (ab102524, Abcam). Briefly, after harvesting, cells were homogenized and incubated with reagents for 60 – 90 minutes at 37°C and absorbance (OD570 nm) was measured in the kinetic mode using BioTek Synergy H1 Plate Reader (Agilent Technologies). Lipase activities were calculated as nmol/min/mL based on the standard curve.

**Immunofluorescence staining and confocal microscopy of fixed cells**

For lysosome staining, cells were seeded on glass slide coverslips within 12-well plates at a density of 80,000, incubated with 75nM LysoTracker DND-99 probes for 1 hr at 37°C and 5% CO2, washed with PBS and fixed with 4% paraformaldehyde in PBS for 10 min at room temperature. For mitochondria staining, cells were incubated 0.2 μM MitoTracker Deep Red (Life Technologies) for 15 minutes before imaging. For other staining, cells were then permeabilized with 0.25% Triton-100 in PBS for 10 min at room temperature, washed with PBS, and incubated with 1% BSA, 22.52 mg/mL glycine in PBST (PBS+ 0.1% Tween 20) for 30 min to block unspecific binding of the antibodies. Cells were incubated with primary antibodies (i.e., LAMP1, PLIN2, LC3B, see Major Resources Tables for details) diluted in 0.1% PBST at 4°C overnight. After 3 washes with PBS, cells were incubated with secondary antibodies diluted in 0.1% PBST for 1 h at room temperature, washed 3 times with PBS, and mounted with ProLong™ Gold Antifade Mountant with DNA Stain DAPI (P36935, Invitrogen). For intracellular lipid accumulation, cells were incubated with 1µM oleate for 24hr, stained with 100 μg/mL Nile red (72485, Sigma) for 30 min, and fixed with 4% paraformaldehyde. All samples were examined under a Zeiss LSM 710 confocal laser-scanning microscope equipped with X63 oil-immersion objective. For quantitative image analysis, randomly chosen non-overlapping fields were scanned using the same setting parameters (i.e., laser power and detector amplification) below pixel saturation. The mean intensity per field was determined using the histogram function in the Zeiss LSM 710 Software, and pixel values above background levels were quantified. All the experiments were repeated at least three times, and representative images are shown. To quantify colocalization (i.e., PLN2/LysoTracker, C16/LysoTracker, C12/LysoTracker, C12/Lamp1, C12/MitoTracker, C12/LDs, C12/LC3B, LC3B/MitoTracker colocalization), randomly chosen non-overlapping fields were scanned and analyzed in Carl Zeiss software AIM using the Pearson’s Correlation Coefficients. All imaging parameters and analyzing settings remained the same for all data acquisition within one experiment.

**Fluorescent FA Pulse-Chase**

Cells were incubated with EMEM with 10% fetal bovine serum, 2mM glutamax, and 500 U/mL penicillin/streptomycin) containing 2 µm BODIPY 558/568 C12 (Red C12, D3835, Invitrogen) or 1 µm BODIPY FL C12 (Green, D3822, Invitrogen) in duplicate wells to allow the fluorescent lipids to incorporate into LDs. After 16 hr, one well was collected as timepoint “HR 0”. The other well was washed three times with PBS, chased with DMEM, and collected after 24 hr as timepoint "HR 24”. At the time of collection, cells were washed twice with PBS, fixed with 4% paraformaldehyde in PBS for 10 min, and permeabilized with 0.25% Triton-100 in PBS for 10 min at room temperature. Mitochondria were labeled with 0.2 μM MitoTracker Deep Red FM for 30 min, lipid droplets were labeled with 1µM BODIPY 493/503 for 30 min, and lysosomes were labeled with 75 nM LysoTracker Red DND-99 for 1 hr. All cells were imaged with Zeiss LSM 710 confocal laser-scanning microscope with X63 oil-immersion objective and analyzed as described in the confocal microscopy section above.

**Live cell imaging**

To directly monitor mitophagy in live cells, we used mKeima-Red-Mito-7 (Plasmid #56018, Addgene). The expressed red fluorescent protein localizes in the mitochondrial inner membrane and has an excitation of ~440 nM (Green), but under acidic pH, its excitation switches to ~550 nM (Red), which indicates the occurrence of mitophagy(3). HepG2 cells were transfected with MitoKeima and siRNAs (Life Technologies) targeting *TMEM55B* (S40499) or scramble siRNA (Scr) using the AMAXA Nucleofector V kit (Lonza, Cologne, Germany). After 48 hours, cells were treated with BSA or 1mM PA for 1 hr and imaged on a spinning disk confocal microscope (Nikon CSU-X1 dual camera platform equipped with Okolab stagetop incubation system and an iXon Ultra 888 EMCCD camera) using a 60X Apo TIRF. For other staining, cells were incubated with 0.2 μM LysoTracker (DND-99, Life Technologies) for 15min, 1 μM BODIPY C12 (Green, Life Technologies) for 30 minutes, 0.2 μM MitoTracker Deep Red (Life Technologies) for 15 minutes, and 5 µM MitoSOX Green for 10 minutes, and imaged on a spinning disk confocal microscope using a 60X or 100X Apo TIRF. Imaging data was collected in 10–20 movies per condition with the same laser power, exposure, and electron-multiplying gain settings for all conditions. Images were subjected to Gaussian filtering and background subtraction in Imaris v 9.6 (Bitplane, Concord, MA). For analysis of trafficking parameters, labeled vesicles were subjected to surface reconstruction using the Surfaces and Tracks modules, and track length, track displacement, and track lifetimes were calculated using Imaris. For analysis of mitochondrial and fatty acid volume, MitoTracker-labeled mitochondria and C12-labeled fatty acids were subjected to surface reconstruction in Imaris, and automated segmentation by color-coding based on the volume of the connected components was used for 3D surface rendering.

**Transmission electron microscopy**

Cells were grown on MatTek glass bottom dishes (P35G-1.5-14-C, MatTek), fixed in 2% glutaraldehyde and 2% paraformaldehyde solution for 24 hrs and washed 3 times for 5 min each in 0.1 M sodium cacodylate buffer, pH 7.4. Samples were then post-fixed in 1% osmium tetroxide with 1.6% potassium ferricyanide (KFECn) in 0.1 M sodium cacodylate buffer for 30 min, and washed 3 times for 15 min each with PBS. Cells were dehydrated in a serial diluted ethanol solution of 30, 50, 70, 90, and 100%, for 10 min each and infiltrated with 50% Epon-Araldite resin (containing benzyldimethylamine accelerator) for 1hr, followed by 100% resin for 1 hr. Excess resin was removed from the MatTek dishes containing cells and polymerized at 60°C for 48 hrs. Using a dissecting blade, cells embedded in resin were mounted on resin-embedded blocks and serial sections of 70-150 nm thickness were cut on a Reichert-Jung Ultracut E microtome and set on 1 x 2-mm slot grids covered with 0.6% Formvar film. Sections were then post-stained with 1% aqueous uranyl acetate for 7 min and lead citrate for 4 min. Samples were imaged on an FEI Tecnai 12 transmission electron microscope equipped with a 2k x 2k CCD camera with a 40 Megapixel/sec readout mode. Images were analyzed using ImageJ software according to the method by Lam et al^1^.

**Measures of mitochondrial levels and oxidative stress**

HepG2 cells were transfected with siRNAs targeting *TMEM55B* or a scrambled control siRNA and plated in 96-well plates at a density of 1x10^e4^ per well. After 48 hours, cells were incubated with 200 nM MitoGreen to stain mitochondria or 5 mM CellRox Green to measure cellular oxidative stress for 30 mins, or 5 µM MitoSox Red in HBSS (14025076, Gibco) for 10 min to measure mitochondria-specific oxidative stress, and 5µg/ml Hoechst for 15 min to stain the nuclei. After 3 washes with PBS, fluorescence levels were quantified with BioTek Synergy H1 Plate Reader (Agilent Technologies). To visualize MitoSox staining, transfected cells were also plated in 6-well plates at a density of 2x10^e5^ per well. After 48 hours, cells were treated with 5 µM MitoSox Red in HBSS (14025076, Gibico) for 10 min and imaged using the Keyence microscope at 20X.

**Measures of mitochondria function**

10,000 HepG2 cells or 7,000 mouse primary hepatocytes were seeded into each well of a Seahorse Bioscience (Agilent, Santa Clara, CA) tissue culture 96-well plate coated with or without poly-D-Lysine and incubated overnight at 37 °C in 5% CO_2_. Cells were washed twice with DMEM assay media (sodium bicarbonate- and glucose-free DMEM supplemented with glutamine and penicillin/streptomycin, pH 7.4) and incubated for 1 hr at 37 °C without CO_2_. Measures of mitochondrial function and oxidation of exogenous and endogenous fatty acids were determined by recording oxygen consumption rates (OCR, pmol/min) on a Seahorse Bioscience XFe96 extracellular flux Analyzer. After measuring basal respiration, the injection of oligomycin was used to measure ATP production (final concentration 2 μM), and the injection of fluorocarbonyl cyanide phenylhydrazone (FCCP) was used to detect maximal respiration (final concentration 2μM). The final injection of antimycin A and Rotenone was to measure spare respiratory capacity and non-mitochondrial respiration. Background values were determined from cell-free wells and subtracted from sample values. Cellular protein levels were determined using a Bradford protein assay. Data were normalized to total protein in each well. To measure fatty acid β-oxidation rates, cells were seeded in XF96 well plates and starved overnight in substrate-limited DMEM supplemented with 0.5 mM of glucose, 1 mM of glutamine, 0.5 mM of carnitine, and 1% FBS. The bovine serum albumin (BSA)-conjugated palmitate (55.8 nM, Seahorse Bioscience) was added to a final concentration of 10 nM, and basal OCR of cells treated with palmitate-BSA or BSA vehicle alone were measured. For cell permeabilization, 1 nmol of Seahorse XF Plasma Membrane Permeabilizer (102504-100, Agilent Technologies) was used.

Mouse liver mitochondria were isolated and quantified as described(4). Briefly, liver tissues were harvested and minced in cold MSHE buffer (210 mM Mannitol, 70 mM sucrose, 5 mM HEPES, 1 mM EGTA, pH 7.2) + 0.5%BSA and homogenized with gentleMACS™ Dissociator (Miltenyi Biotec). The homogenate was centrifuged at 4°C at 800g for 10 min, after which the supernatant was transferred to another tube, and centrifuged again at 4°C at 8000g for 10 min. The pellet was resuspended in 800µl of MSHE buffer + 0.5%BSA and centrifuged again at 4°C at 8000g for 10 min. The pellet was resuspended in 30µl of MAS buffer (220 mM mannitol, 70 mM sucrose, 10 mM KH2PO4, 5 mM MgCl2, 2 mM HEPES, 1.0 mM EGTA, 0.2 % BSA, pH 7.2). Protein concentration was quantified with Pierce™ Bradford Plus Protein Assay Kits (23236, Thermo Scientific). 4 µg of mitochondria were plated/well in a XF96 well plate. OCR was measured after addition of 4 mM ADP (Complex V substrate), oligomycin, FCCP, and antimycin as described above. OCR was normalized per μg mitochondrial protein.

**Animal studies**

Animal studies were performed with approval and in accordance with the guidelines of the Institutional Animal Care and Use Committee at the University of California San Francisco (UCSF). Animals were cared for according to the recommendations of the Panel on Euthanasia of the American Veterinary Medical Association. The animal facility is Association for Assessment and Accreditation of Laboratory Animal Care (AAALAC) approved and is responsible for the health and husbandry of animals. Animal studies comply with the Animal Research: Reporting of In Vivo Experiments (ARRIVE) guidelines. Mice were housed in a climate-controlled Department of Laboratory Animal Medicine facility with a 12-hour light-dark cycle and ad libitum access to food and water.

***Tmem55b* knockout mouse model**

*Tmem55b* floxed mice were purchased from Cyagen Biosciences, who inserted *loxP* sites flanking *Tmem55b* exons 1 to 6 in a C57BL6/N background. The *Tmem55b^fl/+^* mice were backcrossed 7 times to the C57BL/6J strain, and subsequently crossed with Sox-2-Cre mice (Strain #:008454, JAX) on a C57BL/6J background to generate *Tmem55b^+/-^* mice. These mice were intercrossed to generate whole-body *Tmem55b* knockout and littermate control *Tmem55b^+/+^* mice. Mice were confirmed to have a >99% C57BL/6J background using The Jackson Laboratory’s C57BL/6 Substrain Characterization Panel. Primary hepatocytes were isolated from 10-week old male and female mice at the University of California, San Francisco Liver Center using the standard collagenase method(5).

6-week old mice were fed a GAN diet (40% kcal fat, 20% kcal fructose, 2% cholesterol) for 21 weeks, after which mice were euthanized after 4 hr fast. Blood was collected via cardiac puncture, and plasma isolated by centrifugation at 850× g for 15 min at 4 °C. Tissues were flash frozen in liquid nitrogen, and a portion of liver was fixed in 10% formalin, washed with PBS the next day, and stored in 70% ethanol for histology.

**ASO-mediated *Tmem55b* knockdown mouse model**

We have previously described ASO-mediated Tmem55b knockdown mouse model(2). C57BL/6J male mice were purchased from Jackson Laboratory (Bar Harbor, ME). Six-week-old animals were i.p. injected with 25 mg/kg body weight/week of antisense oligonucleotides (ASO) targeting *Tmem55b* or a non-targeting control (Ionis Pharmaceuticals) and fed a GAN Diet (n=10/treatment). Body weight was measured prior to the first ASO injection, and randomized to receive either the *Tmem55b* or NTC ASO. Animals were injected with ASO weekly until sacrifice. After 7, 21, or 29 weeks, mice were fasted for 4 hours, euthanized, and biospecimens collected as described for the *Tmem55b* knockout model.

**Mouse liver immunohistology**

For Oil red O (ORO) staining, mouse liver was embedded in Tissue-Tek, sectioned, and stained in 0.5% Oil Red O solution in propylene glycol for 30 min. The slides were processed for hematoxylin counter staining. For H&E, Sirius Red, and Masson Trichrome staining, a portion of mouse liver was fixed with 10% formalin, embedded in paraffin, and cut into 4-µm sections. Sections were stained with different dyes. All slides were assessed by a pathologist blinded to the sample identity.

**GTT and ITT**

Intraperitoneal glucose tolerance tests (GTT) and insulin tolerance tests (ITT) were performed at weeks 3 and 4 on Western diet and ASO, following 6 h fast(6). Briefly, 2.0 g/kg body weight of glucose or 0.75 U/kg of insulin was injected intraperitoneally, and blood glucose was measured every 15 or 30 mins over 120 min. All blood glucose measurements were performed using a FreeStyle Freedom Lite glucometer (Abbot Diabetes Care, Inc., Alameda, CA).

**Hepatic lipid measurements**

Liver tissues were homogenized with GentleMacs (Miltenyi Biotec Inc. Auburn, CA) and lipids extracted with chloroform-methanol (2:1) according to the Folch method(7). Chloroform extracts were dried under N_2_ gas and resuspended in 200 μl isopropyl alcohol containing 10% Triton X-100. TAG was measured using the L-Type TG M kit (Wako Chemicals, Richmond, VA) following manufacturer’s instructions. Free fatty acid levels were measured in the liver tissue lysate with Non-Esterified Fatty Acid (NEFA) Assay Kits (Wako Chemicals, Richmond, VA).

**Plasma** **aminotransferase measurements**

Mouse plasma aspartate aminotransferase (AST) and alanine aminotransferase (ALT) levels were measured by enzymatic end point measurements using enzyme reagent kits (Ciba-Corning Diagnostics Corporation) in an AMS Liasys 330 Clinical Chemistry Analyzer.

**RNA-sequencing**

Total RNA was extracted from livers of male mice treated with *Tmem55b* (N=4) or non-targeting control (N=4) ASOs and fed a Western diet for 4 weeks, checked for quality on a Bioanalyzer, and made into polyA-selected, strand-specific RNA-seq libraries for 150 bp paired-end sequencing on Illumina NovaSeq machines. Sequence fragments were aligned to the mouse GRCm39 genome and GENCODE transcriptome using STAR 2-pass alignment(8). Fragments aligning to annotated genes were counted using featureCounts(9) and adjusted for library size using DESeq2(10). DESeq2 was used for differential expression analysis, and p-values were adjusted for multiple testing using a false discovery rate (FDR) approach. RNAseq data is available on GEO # GSE273884.

RNAseq data from iPSC-derived hepatocyte like cells was downloaded from GEO (GSE138312), and has been previously described(11). Gene expression count data was adjusted for library size, and variance stabilized to obtain a ~log2 transformation in DESeq2.

1. Medina MW, Bauzon F, Naidoo D, Theusch E, Stevens K, Schilde J, et al. Transmembrane protein 55B is a novel regulator of cellular cholesterol metabolism. Arterioscler Thromb Vasc Biol. 2014;34(9):1917-23.

2. Qin Y, Ting F, Kim MJ, Strelnikov J, Harmon J, Gao F, et al. Phosphatidylinositol-(4,5)-Bisphosphate Regulates Plasma Cholesterol Through LDL (Low-Density Lipoprotein) Receptor Lysosomal Degradation. Arterioscler Thromb Vasc Biol. 2020;40(5):1311-24.

3. Mohamud Yusuf A, Borbor M, Hussner T, Weghs C, Kaltwasser B, Pillath-Eilers M, et al. Acid sphingomyelinase inhibition induces cerebral angiogenesis post-ischemia/reperfusion in an oxidative stress-dependent way and promotes endothelial survival by regulating mitochondrial metabolism. Cell Death Dis. 2024;15(9):650.

4. Norheim F, Hasin-Brumshtein Y, Vergnes L, Chella Krishnan K, Pan C, Seldin MM, et al. Gene-by-Sex Interactions in Mitochondrial Functions and Cardio-Metabolic Traits. Cell Metab. 2019;29(4):932-49 e4.

5. Moldeus P, Hogberg J, Orrenius S. Isolation and use of liver cells. Methods Enzymol. 1978;52:60-71.

6. Johnson AR, Qin Y, Cozzo AJ, Freemerman AJ, Huang MJ, Zhao L, et al. Metabolic reprogramming through fatty acid transport protein 1 (FATP1) regulates macrophage inflammatory potential and adipose inflammation. Mol Metab. 2016;5(7):506-26.

7. Folch J, Lees M, Sloane Stanley GH. A simple method for the isolation and purification of total lipides from animal tissues. J Biol Chem. 1957;226(1):497-509.

8. Dobin A, Davis CA, Schlesinger F, Drenkow J, Zaleski C, Jha S, et al. STAR: ultrafast universal RNA-seq aligner. Bioinformatics. 2013;29(1):15-21.

9. Liao Y, Smyth GK, Shi W. featureCounts: an efficient general purpose program for assigning sequence reads to genomic features. Bioinformatics. 2014;30(7):923-30.

10. Love MI, Huber W, Anders S. Moderated estimation of fold change and dispersion for RNA-seq data with DESeq2. Genome Biol. 2014;15(12):550.

11. Duwaerts CC, Le Guillou D, Her CL, Phillips NJ, Willenbring H, Mattis AN, et al. iPSC-derived hepatocytes from patients with nonalcoholic fatty liver disease display a disease-specific gene expression profile. Gastroenterology. 2021.

**Major Resources Tables**

**Animals (in vivo studies)**

| **Species** | **Vendor or Source** | **Background Strain** | **Sex** |
| --- | --- | --- | --- |
| C57BL/6J | The Jackson Laboratory | C57BL/6J | Male and female |

**Animal breeding**

|  | **Species** | **Vendor or Source** | **Background Strain** | **Other Information** |
| --- | --- | --- | --- | --- |
| **Parent - Male** | Tmem55b^fl/fl^ | Cyagen Biosciences | C57BL/6N |  |
| **Parent - Female** | Tmem55b^fl/fl^ | Cyagen Biosciences | C57BL/6N |  |
| **Parent - Female** | B6.Cg-Edil3Tg(Sox2-cre)1Amc/J | The Jackson Laboratory | C57BL/6J |  |
| **Parent - Male** | Tmem55b^+/-^ | UCSF | C57BL/6J |  |
| **Parent - Female** | Tmem55b^+/-^ | UCSF | C57BL/6J |  |

**Antibodies and Reagents**

| **Name** | **Vendor or Source** | **Catalog #** | **Working concentration** | **Lot # (preferred but not required)** |
| --- | --- | --- | --- | --- |
| Anti-Tmem55b | Proteintech (Rosemont, IL) | 23992 | 2 μg/ml |  |
| Anti-PLIN2 | ABGENT  (San Diego, CA) | AP5118c | 5 μg/ml |  |
| Anti-LC3B | Sigma-Aldrich  (Cambridge, MA) | L7543 | 5 μg/ml |  |
| Anti-p62 | Novus Biologicals (Centennial, CO) | NBP1-48320 | 1 μg/ml |  |
| Anti-CHOP | Cell Signaling Technology (Danvers, MA) | 5554S | 0.186 μg/ml |  |
| Anti-4-HNE | Abcam (Cambridge, MA) | ab46545 | 1 μg/ml |  |
| Anti-Lamp1 | Abcam (Cambridge, MA) | ab24170 | 1 μg/ml |  |
| Anti-GAPDH | Santa Cruz (Dallas, TX) | Sc-166545 | 2 μg/ml |  |
| Goat anti-rabbit IgG H&L (HRP) | Abcam (Cambridge, MA) | ab7090 | 0.2 μg/ml |  |
| Goat anti-rabbit IgG H&L (Alexa Fluor® 488) | Abcam (Cambridge, MA) | ab150077 | 2 μg/ml |  |
| Goat anti-Rabbit IgG (H+L) Cross-Adsorbed Secondary Antibody, Alexa Fluor™ 568 | Invitrogen (Carlsbad, CA) | A-11011 | 2 μg/ml |  |
| BODIPY 493/503 | Invitrogen (Carlsbad, CA) | D3922 | 1 μM |  |
| HCS LipidTOX™ Red neutral  lipid stain | Invitrogen (Carlsbad, CA) | H34476 | 1X |  |
| LysoTracker Red DND-99 | Invitrogen (Carlsbad, CA) | L7528 | 75 nM |  |
| MitoTracker Deep Red FM | Invitrogen (Carlsbad, CA) | M22426 | 100 nM |  |
| MitoGreen | Invitrogen (Carlsbad, CA) | M7514 | 200 nM |  |
| CellRox Green | Invitrogen (Carlsbad, CA) | C10444 | 5 μM |  |
| MitoSox Red | Invitrogen (Carlsbad, CA) | M36008 | 5 μM |  |
| Hoechst | Invitrogen (Carlsbad, CA) | H21486 | 5 μg/ml |  |
| BODIPY™ FL C12 | Invitrogen (Carlsbad, CA) | D3822 | 2 μM |  |
| BODIPY™ 558/568 C12 | Invitrogen (Carlsbad, CA) | D3835 | 1 μM |  |
| BODIPY™ FL C16 | Invitrogen (Carlsbad, CA) | D3821 | 2 μM |  |

**Cultured Cells**

| **Name** | **Vendor or Source** | **Sex (F, M, or unknown)** |
| --- | --- | --- |
| HepG2 | ATCC | M |
| Primary hepatocytes | C57bl/6J | M, F |
| Huh7 | ATCC | M |
