# Supplementary material for: Loss of TMEM55B modulates lipid metabolism through dysregulated lipophagy and mitochondrial function
Source: Cell Death Dis. 2026 Jan 9;17(1):26. doi: 10.1038/s41419-025-08210-x (PMC12789068; doi:10.1038/s41419-025-08210-x)
Supplement: Supplementary file 1 — Supplementary Figures [file 41419_2025_8210_MOESM1_ESM.docx]

**Supplementary Materials**

**Loss of TMEM55B modulates lipid metabolism through dysregulated lipophagy and mitochondrial function**

Yuanyuan Qin^1^, Sheila S. Teker^1^, Nilsa La Cunza^2^, Yao Tong^2^, Elizabeth Theusch^1^, Neil V. Yang^1,3^, Leela Venkatesan^1^, Julia Su^1^, Xuanwen Wang^1^, Ronald M. Krauss^1,4^, Aparna Lakkaraju^2,5^, Aras N. Mattis^4,6^, Marisa W. Medina^1,4,7*^

1. Department of Pediatrics, University of California San Francisco, Oakland, CA 94609
2. Department of Ophthalmology, University of California San Francisco, San Francisco, CA 94143
3. Department of Nutritional Sciences and Toxicology, University of California Berkeley, Berkeley, CA 94720
4. Liver Center, University of California San Francisco, San Francisco, CA
5. Pharmaceutical Sciences and Pharmacogenomics Graduate Program, University of California San Francisco, San Francisco, CA 94143
6. Department of Pathology, University of California San Francisco, San Francisco, CA 94143
7. Institute for Human Genetics, University of California San Francisco, San Francisco, CA 94143

*corresponding author

**Supplementary Fig. S1.**  **A**) Study design of in vivo Tmem55b knockdown. Six-week old male C57BL/6J mice were treated with an ASO against Tmem55b (Tmem55b-ASO) or a non-targeting control (NTC) at a dose of 25 mg/kg body weight/week and fed a Western diet (0.2% cholesterol, 42% fat) for 6 weeks. **B**) Glucose tolerance test (GTT) and insulin tolerance test (ITT) were performed. N=5/ASO. **C**) Schematic showing the experimental design to test the long-term effect of Tmem55b knock-down in Western diet-fed male C57BL/6J mice treated with Tmem55b-ASO or NTC-ASO for 21 or 29 weeks. **D**) RNAseq results from the liver of Tmem55b-ASO and non-target ASO treated male mice. Six-week old male C57BL/6J mice were treated with an ASO against Tmem55b (Tmem55b-ASO) or a non-targeting control (NTC) at a dose of 25 mg/kg body weight/week and fed a Western diet (0.2% cholesterol, 42% fat) for 4 weeks. Total RNA was extracted from mouse livers (n=4/ASO), checked for quality on a Bioanalyzer, and made into polyA-selected, strand-specific RNA-seq libraries for 150 bp paired-end sequencing on Illumina NovaSeq machines. DESeq2 was used for differential expression analysis, and p-values were adjusted for multiple testing using a false discovery rate (FDR) approach. 68 of the 17,903 genes tested were significantly differentially expressed at an FDR=5% (38 upregulated and 31 downregulated. Purple color dots indicate genes (*Prkn, Immp2i*, and *Sugct*) that regulate mitochondrial function, while blue color dots indicate upregulated genes involved in cell adhesion (*Flrt2*, *Egfl7*, *Col8a1*, *Pkp3*, *Pcdh17, Mcam*). **E**) Schematic of the *Tmem55b* knockout allele. We created a *Tmem55b* KO model by inserting loxP sites flanking Tmem55b exons 1 to 6 in a C57BL/6N background. The Tmem55b^fl/+^ mice were backcrossed 7 times to the C57BL/6J strain and subsequently crossed with Sox-2 mice to generate whole-body Tmem55b^-/-^ (KO) mice on a >99% C57BL/6J background. **F**) Schematic showing the experimental design of six-week-old male and female wildtype and *Tmem55b* KO mice fed a GAN diet for 12 weeks.

**Supplementary Fig. S2.** Phenotypes of female and male WT and *Tmem55b* KO mice. Mice were fed a GAN diet starting at 6-week-old for 12-weeks (female mice N=12 WT, N=13 KO; male mice N=13 WT, N=9 KO). Body weight, liver weight, and fasting glucose were measured in both female (**A**) and male mice (**B**). Plasma AST, ALT, and AST/ALT were detected by Liasys 330 Clinical Chemistry Analyzer in female (**A**) and male (**B**) mice. Liver histology of female (**A**) and male (**B**) mice was evaluated by a blinded pathologist. Scale bar=100 µm. (For histology, female mice N=5 WT, N=12 KO; male mice N=7 WT, N=11 KO). For the animal studies, quantified results are presented as mean ± s.e.m. *p<0.05, **p<0.01, ****p<0.0001 vs. WT by Student’s t-test.

**Supplementary Fig. S3.** Plasma lipid phenotypes of female (**A**) and male (**B**) WT and *Tmem55b* KO mice. Mice were fed a GAN diet starting at 6 weeks old for 12 weeks. Plasma triglyceride (TG), total cholesterol (TC), HDLc (high-density lipoprotein cholesterol) were quantified with AMS Liasys 330 Clinical Chemistry Analyzer, and non-HDLc was calculated by subtraction of HDLc from TC (female mice N=12 WT, N=13 KO; male mice N=13 WT, N=9 KO). Quantified results are presented as mean ± s.e.m. *p<0.05, **p<0.01 vs. WT by Student’s t-test.

**Supplementary Fig. S4.** Human hepatoma cell lines were transfected with TMEM55B or Scr siRNAs for 48hr. **A)** *TMEM55B* transcript levels were quantified with qPCR in HepG2 cells (N=3 biological replicates). **B)** HepG2 transfected cells were stained with LysoTracker and visualized using confocal microscopy for quantification of lysosome number and area per cell. N=38 cells Scr siRNA, N=49 cells *siTMEM55B* siRNA. **C**) Lysosome speed, track length, and duration were measured using live cell imaging of siRNA-transfected HepG2 cells after incubation with 1 mM palmitate treatment for 1 hr. N=6 non-overlapping images/siRNA. **D**) TMEM55B knockdown showed no effect on cytosolic lipase activity in either HepG2 or Huh7 cells using the Lipase Assay Kit from Abcam N=3 biological replicates. **E**) Transfected HepG2 cells were incubated with or without 100nM Bafilomycin A1 for 2 hours, and LC3B protein levels were measured and quantified by immunoblot. N=5 biological replicates. **F**) HepG2 cells were transfected with TMEM55B or Scr siRNAs. After 48 hr, HepG2 cells were incubated with 1mM oleate for 16 hr, labeled with anti-PLIN2 and LysoTracker and examined by confocal microscopy (63X, scale bars=10 µm), N=28 cells Scr siRNA, N=39 cells TMEM55B siRNA. **G**) Transfected HepG2 cells were incubated with 2 µm of green BODIPY™ FL C12-FA overnight, stained with anti-LAMP1 antibody for 1 hr at room temperature, followed by 2^nd^ Goat anti-rabbit IgG (H+L) Alexa Fluor 568 for 1 hr, and examined by confocal microscopy (63X, scale bars=10 µm). N=32 cells Scr siRNA, N=40 cells *siTMEM55B* siRNA. **H**) Transfected Huh7 cells were incubated with 2 µm of green BODIPY™ FL C16-FA overnight, stained with LysoTracker for 1 hr, and examined by confocal microscopy (63X, scale bars=20 µm). N=66 cells Scr siRNA, N=46 cells *siTMEM55B* siRNA. Representative images are shown here. Results are presented as mean ± s.e.m. *p<0.05 **p<0.01, ****p<0.0001 vs. Scr siRNA by Student’s t-test.

**Supplementary Fig. S5. A**) Schematic representation of the FA pulse-chase assays. HepG2 cells were transfected with TMEM55B and Scr siRNAs, pulsed with Red C12-FA (1µM) overnight, washed, and chased with substrate-limited DMEM supplemented with 0.5 mM of glucose, 1 mM of glutamine, 0.5 mM of carnitine, and 1% FBS for 24 hours. Cells were then incubated with 1 µM BODIPY 493/503 to label lipid droplets and imaged with Zeiss 710 confocal microscope. **B**). Representative images and quantitation before and after the chase. N=33 cells Scr siRNA, N=46 cells TMEM55B siRNA at Hr0; N=29 cells Scr siRNA, N=52 cells TMEM55B siRNA at Hr24. Results are presented as mean ± s.e.m. **p<0.01 vs Hr0; ####p<0.0001 vs Scr by two-way ANOVA.

**Supplementary Fig. S6.** The effects of TMEM55B deficiency on mitochondria. Cellular bioenergetics was measured and calculated using Seahorse Bioscience in **A**) primary hepatocytes from female WT and *Tmem55b* KO mice (N=32 technical replicates/genotype), **B**) HepG2 cells transfected with siRNAs targeting *TMEM55B* or scrambled siRNA (N=6 technical replicates/siRNA). **C**) HepG2 cells were transfected with siRNAs targeting TMEM55B or scrambled siRNA. After 48 hr, cells were treated with XF Plasma Membrane Permeabilizer (PMP), and oxygen consumption rate (OCR) was measured by Seahorse XF Analyzer with and without the addition of 40 µm palmitate-coA. N=11 technical replicates/siRNA. **D**) C57BL/6J male mice were fed a GAN diet (40% kcal fat, 20% kcal fructose, 2% cholesterol) for 21 weeks starting at 6 weeks of age. The animals were i.p. injected with 25 mg/kg body weight/week of *Tmem55b* or NTC ASOs (Ionis Pharmaceuticals). Mitochondria were isolated from the liver tissues of 27-week-old male mice, and OCR was measured by Seahorse XF Analyzer. N=12 technical replicates/ASO. **E**) After transfection, HepG2 cells were treated with 200nM MitoGreen for 30 min, and fluorescence levels were quantified with BioTek Synergy H1 Plate Reader. N=7 technical replicates Scr siRNA, N=15 technical *TMEM55B* siRNA. **F**) After transfection, HepG2 cells were labeled with anti-LC3B and MitoTracker Deep Red and examined by confocal microscopy (63X, scale bars=10 µm). N=34 cells Scr siRNA, N=40 cells TMEM55B siRNA. **G)** HepG2 cells transfected with siRNAs targeting *TMEM55B* or scrambled siRNA. After 48 hours, cells were treated with 500 nM CellRox, stained with Hoechst, and fluorescence levels were quantified on the BioTek Synergy H1 Plate Reader. N=11 Scr siRNA, N=14 *TMEM55B* siRNA technical replicates. **H**) Mitochondrial oxidative stress was measured by MitoSox in siRNA transfected HepG2 cells (Scale bars, 100 µm). N=20 images non-overlapping Scr siRNA, N=24 images non-overlapping TMEM55B siRNA. **I)** Primary hepatocytes were isolated from WT and *Tmem55b* KO mice, incubated with or without 20 μM Urolithin A (UA) for 16 hours, treated with 1mM palmitate (PA) for 1 hour, stained with CellRox Green, and quantified by FACS. N=3 technical replicates/genotype. Results are presented as mean ± s.e.m. **p<0.01, ***p<0.001, ****p<0.0001 vs Scr or NTC by Student’s t-test or One-way ANOVA.

**Supplementary Fig. S7.** HepG2 cells were transfected with MT-mKeima-Red plasmid and siRNAs targeting *TMEM55B* or scramble siRNA (Scr) using the AMAXA Nucleofector. After 48 hours, cells were treated with BSA or 1mM PA for 1hr and imaged on Nikon spinning disk confocal microscope at 60X in five randomly selected non-overlapping areas. Four images/treatment are shown here, with the fifth image/treatment shown in the main figures (Figure 4E).

**Supplementary Fig. S8.** TMEM55B knockdown led to increased FA uptake and accumulation. **A**) Protein levels of CD36 in the livers between Tmem55b-ASO and NTC-ASO-treated males were quantified by Western blot (N=6/ASO). **B**) Huh7 cells were transfected with siRNAs targeting TMEM55B (siTMEM55B) or scrambled (Scr) siRNA. After 48 hr, *TMEM55B* transcript levels were measured by qPCR (N=3 biological replicates). After transfection, HepG2 cells were incubated with 2μM Green BODIPY C12-FA for16 hr (**C**) (N=6 technical replicates/siRNA) and chased with substrate-limited medium for 24 hr (**D**) (N=3 biological replicates) before fluorescence was quantified by flow cytometry. **E**) Primary hepatocytes were isolated from WT and *Tmem55b* KO female mice, incubated with or without 20uM Urolithin A (UA) for 16 hours, treated with BSA or 1mM palmitate (PA) for 1 hour, stained with BODIPY 493/503, and quantified by FACS. N=2 technical replicates/siRNA in BSA and PA; N=1/siRNA in PA+UA). **F**) *JIP4* transcript levels were quantified by qPCR in HepG2 control (Ctl) and JIP4 knockout (KO) cell line. N=3 technical replicates/genotype. **G)** *JIP4* transcript levels were quantified by qPCR in HepG2 cells after transfection with scrambled siRNA (Scr) and siRNA targeting JIP4 (siJIP4). N=3 technical replicates/siRNA. Results are presented as mean ± s.e.m. **p<0.01, ***p<0.001, ****p<0.0001 v.s Scr siRNA or NTC ASO by Student’s t-test or One-way ANOVA.

**Supplementary Fig. S9.** The design of single gRNA targeting *JIP4* (VB211203-1192efm (**A**) and non-targeting gRNA as a negative control (VB220208-1241bcc (**B**) using VectorBuilder.

**Supplementary Fig. S10.**  Complete western blot images of cropped blots included within the primary figures. Areas of each blot contained in the quantifications shown in the primary figures are enclosed within the green boxes. **A**) Tmem55b and (**B**) Gapdh protein levels in the livers of wildtype and TMEM55B knockout male mice as shown in Fig 1E. **C**) CHOP, (**D**) 4-HNE, and (**E**) β-actin protein levels in the liver of WT and *Tmem55b* KO mice as shown in Fig. 1G. **F**) LC3B, (**G**) p62, and (**H**) Gapdh protein levels in the livers of WT and *Tmem55b* KO male mice as shown in Fig 2D. **I**) CD36 and (**J**) Gapdh protein levels in the livers of ASO-NTC and ASO-Tmem55b treated male mice as shown in Supplementary Fig. S8A.

**Supplementary Fig. S11.**  Original Western blot images of HepG2 cells as shown in Supplementary Fig. S4D.
